# Small-molecule TFEB pathway agonists that ameliorate metabolic syndrome in mice and extend *C*. *elegans* lifespan

**DOI:** 10.1038/s41467-017-02332-3

**Published:** 2017-12-22

**Authors:** Chensu Wang, Hanspeter Niederstrasser, Peter M. Douglas, Rueyling Lin, Juan Jaramillo, Yang Li, Nathaniel W. Oswald, Anwu Zhou, Elizabeth A. McMillan, Saurabh Mendiratta, Zhaohui Wang, Tian Zhao, Zhiqaing Lin, Min Luo, Gang Huang, Rolf A. Brekken, Bruce A. Posner, John B. MacMillan, Jinming Gao, Michael A. White

**Affiliations:** 1 Department of Pharmacology, Simmons Comprehensive Cancer Center, University of Texas Southwestern Medical Center, 5323 Harry Hines Boulevard, Dallas, TX 75390 USA; 2 Department of Cell Biology, University of Texas Southwestern Medical Center, 5323 Harry Hines Boulevard, Dallas, TX 75390 USA; 3 Department of Biochemistry, University of Texas Southwestern Medical Center, 5323 Harry Hines Boulevard, Dallas, TX 75390 USA; 4 Department of Molecular Biology, University of Texas Southwestern Medical Center, 5323 Harry Hines Boulevard, Dallas, TX 75390 USA; 5 Department of Surgery, University of Texas Southwestern Medical Center, 5323 Harry Hines Boulevard, Dallas, TX 75390 USA

## Abstract

Drugs that mirror the cellular effects of starvation mimics are considered promising therapeutics for common metabolic disorders, such as obesity, liver steatosis, and for ageing. Starvation, or caloric restriction, is known to activate the transcription factor EB (TFEB), a master regulator of lipid metabolism and lysosomal biogenesis and function. Here, we report a nanotechnology-enabled high-throughput screen to identify small-molecule agonists of TFEB and discover three novel compounds that promote autophagolysosomal activity. The three lead compounds include the clinically approved drug, digoxin; the marine-derived natural product, ikarugamycin; and the synthetic compound, alexidine dihydrochloride, which is known to act on a mitochondrial target. Mode of action studies reveal that these compounds activate TFEB via three distinct Ca^2+^-dependent mechanisms. Formulation of these compounds in liver-tropic biodegradable, biocompatible nanoparticles confers hepatoprotection against diet-induced steatosis in murine models and extends lifespan of *Caenorhabditis elegans*. These results support the therapeutic potential of small-molecule TFEB activators for the treatment of metabolic and age-related disorders.

## Abstract (web summary)

Activation of autophagy, via the transcription factor TFEB, is a promising strategy to treat metabolic diseases. Here, the authors report three novel classes of small molecules that promote TFEB nuclear translocation, and provide evidence for the therapeutic efficacy of these compounds in mice and worms.

## Introduction

Autophagosome-lysosome biogenesis is a major adaptive catabolic process that both generates nutrients and energy during starvation and maintains homeostasis under nutrient-rich conditions. Impairment of this process is mechanistically associated with metabolic disorders and ageing. In metabolic syndromes such as obesity^1, 2^ and fatty liver disease^2, 3^, excess nutrients increase demand for degradative autophagy-lysosome machinery and challenge the adaptive response capacity. Ineffective digestion of macromolecules (lipids, proteins, and glycogen) and impaired organelle turnover compromise metabolic activity at the tissue level, provoke intracellular stresses, and exacerbate collateral defects in insulin action or other metabolic pathologies. During ageing and within age-related disorders^4–6^, a steady decline in productive autophagy impairs clearance of defective organelles leading pathological accumulation of pro-apoptotic factors and reactive oxygen species (ROS). Therefore, pharmacological interventions that enhance lysosome function are emerging as a promising strategy to ameliorate metabolic symptoms and promote longevity.

The transcription factor EB (TFEB) positively modulates lipid catabolism^7^ and promotes longevity^8^. This is a consequence of direct induction of the ‘‘coordinated lysosomal expression and regulation’’ (CLEAR) network^9^, which includes genes that control autophagy, lysosome biogenesis, and lipolysis^7, 10–13^. TFEB belongs to microphthalmia-associated transcription factor (MITF)/transcriptional factor E (TFE) family (MiT) of basic helix-loop-helix leucine zipper transcriptional factors that includes MITF, TFEB, transcription factor E3 (TFE3) and transcription factor EC (TFEC)^14–16^. TFEB and TFE3 share extensively overlapping functions and regulatory mechanisms^10, 17–19^. Notably, TFEB/TFE3 overexpression in the liver is sufficient to mimic many transcriptional changes that occur during starvation^7, 20^. In *Caenorhabditis elegans (C*. *elegans)*, overexpression of the TFEB homolog HLH-30 also increases lifespan, likely through induction of macroautophagy^8^. Consequently, TFEB/TFE3 agonists are of interest for potential therapeutic intervention for some metabolic disorders and/or ageing.

Here, we establish a nanotechnology-enabled screening strategy to identify small-molecule TFEB agonists that shift maturation of autophagosomes to degradative autolysosomes. From a chemical library of approximately 15,000 compounds, we identified a small cohort of FDA-approved drugs, marine-derived natural product fractions and synthetic small molecules that promote lysosomal maturation via TFEB activation. Mechanism of action and preclinical proof of concept studies were completed for three molecules: digoxin, ikarugamycin, and alexidine dihydrochloride, which engaged TFEB activation mechanisms via three distinct Ca^2+^ sources and Ca^2+^-sensing pathways. All three compounds improved lipid metabolism and overcame insulin-resistance in a mouse model of diet-induced fatty liver disease. Furthermore, ikarugamycin was delivered to nematode cultures, and resulted in activation of the nematode TFEB ortholog and lifespan extension. These molecules represent leads for development of therapeutic strategies directed against metabolic syndromes, ageing, and age-related disorders, and can serve as pharmacological tools to help provide new biological insights on Ca^2+^-dependent regulation of cell regulatory systems that impact lysosomes/autophagosome functions.

## Results

### A UPS-enabled high-throughput screen for TFEB agonists

**Fig. 1:**
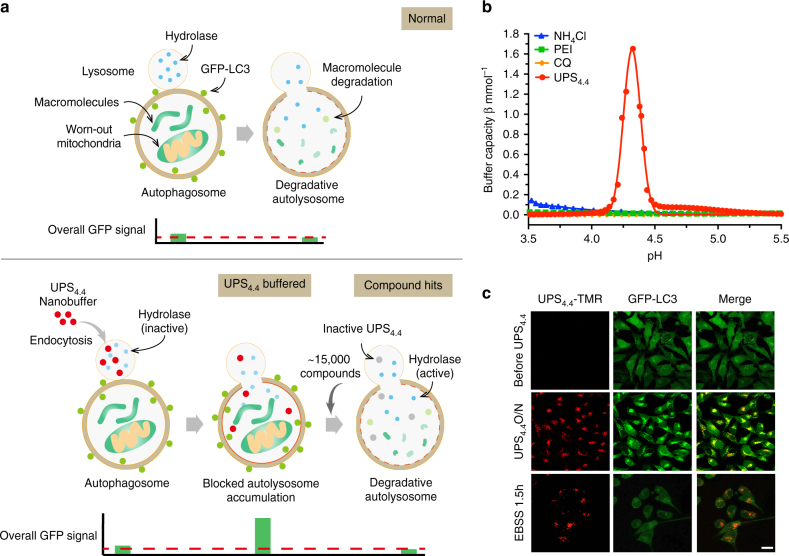
A UPS-enabled compound screen design that allows for discovery of agents that promote maturation of autophagosomes to degradative autolysosomes. **a** A schematic showing the design of a quantitative high-throughput cell-based assay for agents that promote maturation of autophagosomes to degradative autolysosomes. Under normal conditions, GFP-LC3 is degraded in autolysosomes resulting in a mild decrease in total fluorescent intensity of GFP signals. UPS_4.4_-buffered autolysosomes have a pH environment that is not optimal for hydrolase activation. In consequence, GFP-LC3 accumulates in the cytosol resulting in a detectable increase in GFP fluorescence intensity as compared to controls. Compounds that promote autophagic flux and lysosomal function overcome the buffering effect of UPS_4.4_ and transition the accumulated defective autolysosomes into degradative autolysosomes. This results in a reduction in GFP fluorescence intensity that is reproducibly detectable in a high-throughput setting. **b** Buffer capacity (β) of UPS_4.4_, NH_4_Cl, chloroquine (CQ) and polyethylenimine (PEI) was plotted as a function of pH. **c** Representative images showing the effect of UPS_4.4_-TMR treatment and a subsequent nutrient-starvation on GFP-LC3 puncta accumulation. Scale bar, 20 μm

**Fig. 2:**
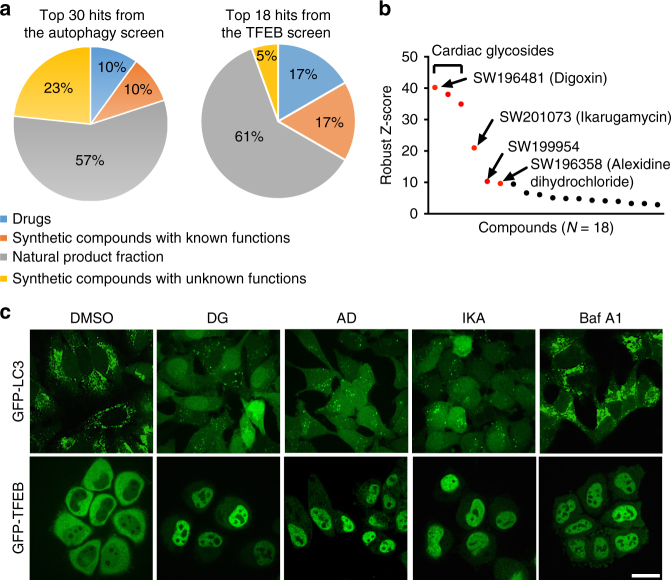
High-throughput screen for small-molecule agonists of TFEB. **a** Pie charts showing the composition of the top 30 hits from the autophagy screen (left) and the top 18 hits from the TFEB screen (right). The top 18 hits include 3 FDA-approved drugs (digoxin, proscillaridin A, and digoxingenin), 11 natural product fractions and 4 synthetic small molecules (including alexidine dihydrochloride and cycloheximide). **b** Robust *Z*-score plot of the top 18 chemicals in the TFEB screen that overlap with the top 30 hits from the autophagy screen. **c** Representative images of GFP-LC3 and GFP-TFEB HeLa cells treated with 370 nM DG, 3.3 μM AD, and IKA and 50 nM bafilomycin A1 (Baf A1). GFP-LC3 HeLa cells were pretreated with UPS_4.4_ prior to a 4 h compound exposure. Baf A1, which blocks autolysosomal degradation through inhibition of vacuolar ATPases, was used as a negative control. In GFP-TFEB HeLa cells, the same concentration of compounds was used without UPS_4.4_, while Baf A1 was used as a positive control. Scale bars, 20 μm

A key functional consequence of TFEB activation is enhanced clearance of deleterious macromolecules and organelles through autophagic and lysosomal degradation. Therefore, in order to identify new chemical probes that promote TFEB activity, we first designed a quantitative high-throughput cell-based assay for agents that promote maturation of autophagosomes to degradative autolysosomes (Fig. 1a). This was enabled by a fine-scale UPS nanobuffer library^21^, where each micelle nanoparticle is composed of ~ 800 copolymer chains with a total of 60,000 ionizable tertiary amine groups^22^. At specific transition pH, each micelle undergoes a phase transition, or de-micellization, which renders a strong buffering capacity within 0.3 pH range. This unique pH cooperative buffer effect was implemented to clamp the luminal pH of endocytic organelles at distinct maturation stages^23^. Among the UPS nanoprobes, UPS_4.4_ specifically arrests lysosomal acidification at pH ~ 4.4 (Fig. 1b and Supplementary Fig. 1a), thereby inhibiting lysosomal/autolysosomal hydrolysis of macromolecules without inhibiting the regulation of mammalian target of rapamycin complex 1 (mTORC1) on lysosomes^23^. Turnover of microtubule-associated protein 1A/1B light chain 3 (LC3), the ortholog of yeast autophagy-related protein 8 (ATG8), was selected as a quantitative measure of lysosome maturation. LC3-II (lipid-modified form of LC3) coats the double membrane structures that encapsulate material that is delivered to autolysosomes, and is itself degraded within those compartments^24^. Thus, GFP-LC3 fusion proteins are commonly employed as live-cell markers for monitoring autophagic flux. UPS_4.4_ exposure was sufficient to induce a remarkable accumulation of cytoplasmic GFP-LC3 puncta (Fig. 1c and Supplementary Fig. 1b) that colocalized with fluorescently labeled UPS_4.4_ nanoprobes as well as lysosomal marker LAMP1 (UPS_4.4_-TMR, Supplementary Fig. 1c, d). Moreover, nutrient restriction was sufficient to promote vacuolar ATPase-dependent clearance of these puncta within 90 min, with consequent reduction of GFP fluorescence intensity (Fig. 1c and Supplementary Fig. 1e, f), resulting in an almost binary ON/OFF signal that can be accurately measured by a microplate reader in a high-throughput screen setting. Blocking autolysosomal functions by UPS_4.4_ resulted in a considerable increase in GFP-LC3 puncta accumulation and fluorescence intensity (six-fold) over starvation-induced autophagic degradation. This is in contrast to a maximal 1.5-fold fed versus starvation signal in the absence of UPS_4.4_ (Supplementary Fig. 1g, h). The ready clearance of UPS_4.4_–induced accumulation of GFP-LC3 indicated the presence of a dynamic cell biological system, which promotes robust induction of autophagosome maturation in response to nutrient starvation, and is amenable to chemical interrogation. We, therefore, leveraged this readout to evaluate ~ 15,000 chemical entities for cellular activity that mimics nutrient starvation (Supplementary Fig. 2a–d). Thirty (out of 80) primary hits were confirmed by independent analyses (Fig. 2a and Supplementary Fig. 2b, e). We next evaluated these compounds for effects on TFEB activity under nutrient replete culture conditions. Transcriptional competence of TFEB is modulated by physical compartmentalization in the cytoplasm (off-state) versus the nucleus (on-state). Chemically induced TFEB nuclear translocation was, therefore, monitored using the fluorescent intensity ratio of nuclear versus cytoplasmic GFP-TFEB (Supplementary Fig. 2f–h). The quantitative robustness of these assays was indicated by a low coefficient of variance (% CV) and a high Z-factor calculated for the neutral control condition (Supplementary Fig. 2i). The top scoring hits included three cardiac glycosides (digoxin, proscillaridin A, and digoxigenin), two natural-product fractions (SW201073 and SW199954), and the synthetic small-molecule alexidine dihydrochloride (Fig. 2a, b and Supplementary Fig. 2j). Structure determination revealed the bioactive component of SW201073 is identical to ikarugamycin (Supplementary Fig. 2k, l), a macrocyclic antibiotic first isolated from *Streptomyces phaeochromogenes*^25^. We chose to further pursue mechanism of action studies with digoxin (DG), alexidine dihydrochloride (AD), and ikarugamycin (IKA), as they were the top-ranking representatives from three distinct categories of chemical compounds.

Consistent with the primary screen results, all three compounds promoted autophagic flux and activated TFEB in a dose-dependent manner as indicated by clearance of UPS_4.4_–dependent accumulation of GFP-LC3 puncta; increased autophagic flux (conversion of LC3-I to LC3-II); turnover of the long-lived autophagy adaptor protein p62/SQSTM1^26^; and translocation of GFP-TFEB from cytosol to nucleus (Fig. 2c and Supplementary Fig. 2m–r). The activity of DG was consistent with isolation of this compound as a hit in a Prestwick-library-focused screen for compounds that promote bulk autophagy^27^. The EC50 of these compounds for promotion of p62/SQSTM1 clearance was generally higher than corresponding TFEB nuclear accumulation EC50s consistent with time and signal delta between TFEB activation and transcription-dependent autophagy induction (Supplementary Fig. 2m, p, q). Activation of TFEB is known to induce the expression of numerous lysosomal and autophagic genes to promote lysosomal biogenesis and maturation^9, 11^. Indeed, all three compounds induced the expression of TFEB target genes^7^ (Supplementary Fig. 2s), and accelerated cellular endosomal maturation rates as indicated by accumulation of acidified organelles (Supplementary Fig. 2t) and activation of cathepsin B (Supplementary Fig. 2u). Furthermore, short-interfering RNA (siRNA)-mediated TFEB depletion was sufficient to inhibit the capacity of the compounds to induce autophagic flux or activate TFEB target genes (Supplementary Fig. 2v, x). Depletion of both TFEB and its homolog, TFE3, further hindered cellular responses to all three compounds (Supplementary Fig. 2 w, x).

### Distinct engagement of mTORC1 by TFEB agonists

**Fig. 3:**
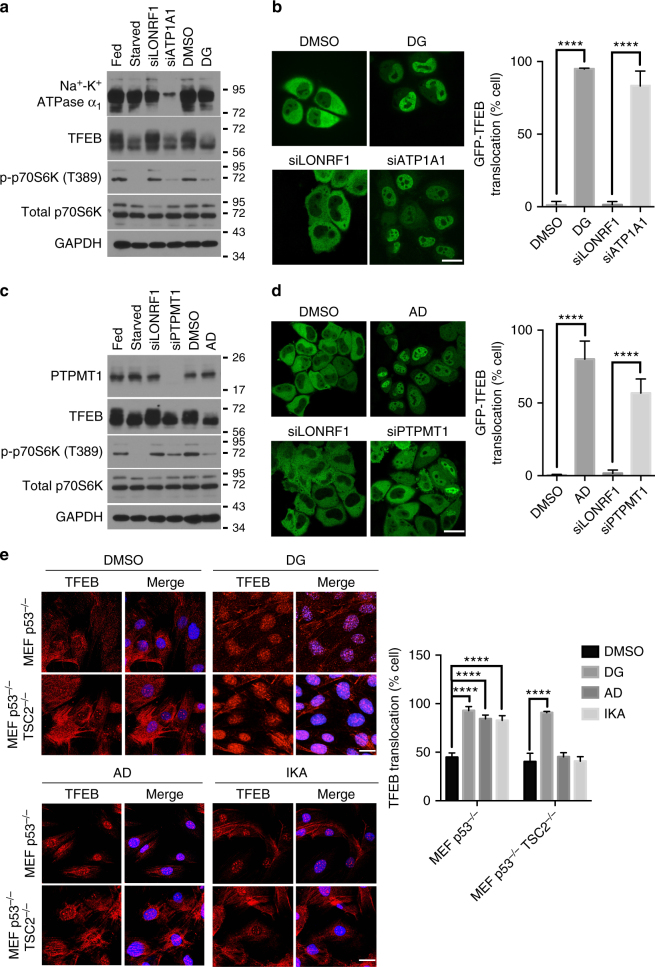
Target-dependent activation of TFEB and inhibition of mTORC1 by DG, AD, and IKA. **a**–**d** siRNA-mediated depletion of the α_1_ subunit of Na^+^-K^+^-ATPase (**a**) and PTPMT1 (**c**) mimics the molecular weight shift of TFEB and inhibition of mTORC1 as seen in the immunoblots of DG-treated (**a**) and AD-treated (**c**) and nutrient-deprived cells (positive controls). Representative confocal images of GFP-TFEB HeLa cells treated with DG, siATP1A1 (**b**) AD, siPTPMT1 (**d**) and their corresponding controls. siRNA against LON peptidase N-terminal domain and ring finger 1 (LONRF1) was used as a negative control siRNA. The graphs represent the percentage of cells with GFP-TFEB translocation under these conditions (mean ± s.d. for *n* = 3 independent experiments, ****p < 0.0001 by two-way ANOVA). Scale bar, 20 μm. **e** To test if the compound-mediated inhibition of mTORC1 was dependent on the well-known negative regulator TSC2, we employed *p53*^*−/−*^ and *p53*^*−/−*^, *TSC2*^*−/−*^ mouse embryonic fibroblasts (MEFs). Endogenous TFEB in cells treated with 370.4 nM DG, 3.3 μM AD, or IKA or DMSO was examined by immunofluorescent staining. TFEB translocation percentage was quantified in the bar graph (mean ± s.d. for *n* = 3 independent experiments, ****p < 0.0001 by two-way ANOVA). Scale bars, 20 μm

Known direct molecular targets of DG and AD in cells are the Na^+^-K^+^ ATPase α_1_ subunit (encoded by *ATP1A1*)^28^ and the protein tyrosine phosphatase mitochondrial 1 (PTPMT1)^29^, respectively. siRNA-mediated depletion of these targets recapitulated GFP-TFEB nuclear translocation in nutrient replete culture conditions, suggesting DG (Fig. 3a, b) and AD (Fig. 3c, d) engage TFEB through their reported cellular targets. The activity of TFEB is known to be directly regulated by the kinase mTORC1 and the phosphatase calcineurin, where mTORC1 directly phosphorylates TFEB S142 and S211 to promote cytosolic sequestration via phospho-serine-dependent interaction with 14-3-3 proteins^11, 13, 17^ while calcineurin dephosphorylates TFEB and promotes its nuclear localization. To begin to parse how these targets engage TFEB, we first examined mTOR pathway activity. Exposure to all three compounds as well as depletion of the known compound targets, Na^+^-K^+^ ATPase α_1_ subunit or PTPMT1, resulted in detectable dephosphorylation of the mTORC1 substrate p70 S6 kinase (p70S6K) under nutrient replete culture conditions, consistent with an inhibition of mTORC1 activity that would normally occur in response to nutrient starvation (Fig. 3a, c**)**. However, inhibition of mTORC1 and activation of TFEB by DG and proscillaridin A (PA, one of the cardiac glycoside hits) was independent of the nutrient-responsive mTOR inhibitory component tuberous sclerosis 2 (TSC2) (Fig. 3e and Supplementary Fig. 3a–c). By contrast, the impact of AD and IKA on mTORC1 pathway activity was TSC2-dependent (Fig. 3e and Supplementary Fig 3d). This suggested distinct mechanisms of engagement of mTORC1 and TFEB by DG, AD, and IKA.

### Disparate Ca^2+^-dependent mechanisms mediate AD, DG, and IKA induction of TFEB

**Fig. 4:**
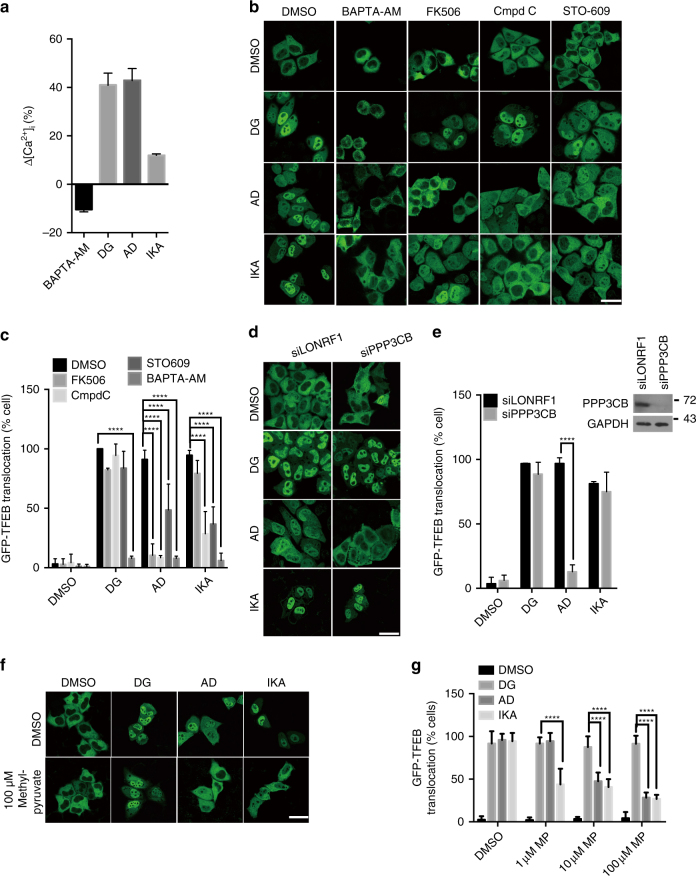
Engagement of distinct Ca^2+^ pathways by small-molecule agonists of TFEB. **a** Intracellular Ca^2+^ concentration was measured in wild-type HeLa cells treated with 5 μM BAPTA-AM, 370 nM DG, 3.3 μM AD, and IKA using Fura-2-AM, and the concentration difference between compound-treated and DMSO-treated cells was normalized to the Ca^2+^ concentration in DMSO-treated cells (*n* = 3 independent experiments). **b** Confocal images of GFP-TFEB HeLa cells treated with 5 μM BAPTA-AM, 5 μM FK506, 10 μM compound C (Cmpd C) and 25 μM STO-609 together with 370 nM DG, 3.3 μM AD, and IKA for 4 h. Scale bar, 20 μm. **c** The graph represents the percentage of cells with GFP-TFEB translocation in **b** (mean ± s.d. for *n* = 3 independent experiments, ****p < 0.0001 by two-way ANOVA). **d** Representative images of GFP-TFEB HeLa cells treated with DG, AD, and IKA together with control siRNA (siLONRF1) treatment, siRNA-mediated inhibition of PPP3CB (siPPP3CB) or in combination with 5 μM calcineurin inhibitor FK506 for 4 h. Scale bar, 20 μm. **e** The graph represents the percentage of cells with GFP-TFEB translocation in **d** (mean ± s.d. for *n* = 3 independent experiments, ****p < 0.0001 by two-way ANOVA). Inset shows the immunoblot of HeLa cells treated with siLONRF1 or siPPP3CB. **f** Cell-permeable pyruvate can reverse the TFEB nuclear translocation induced by AD and IKA, but not DG. Scale bar, 20 μm. **g** The graph represents the percentage of cells with GFP-TFEB translocation in **f** (mean ± s.d. for *n* = 3 independent experiments, ****p < 0.0001 by two-way ANOVA)

A key TFEB activation mechanism is calcium/calmodulin-dependent dephosphorylation of TFEB (S142) by the calcineurin protein phosphatase^12^. DG, AD, or IKA exposure at ~ TFEB_EC90_ was sufficient to induce accumulation of cytosolic calcium as indicated by the quantification of Fura-2 imaging (Fig. 4a), and Ca^2+^ chelation by BAPTA-AM was sufficient to block TFEB activation and reverse mTORC1 inhibition by all three compounds (Fig. 4b and Supplementary Fig. 4a). Direct inhibition of calcineurin with FK506^30, 31^, cyclosporine A (CsA)^30, 31^, or RNAi-mediated depletion of the calcineurin catalytic subunit (PPP3CB) was also sufficient to block TFEB activation by AD, suggesting small-molecule inhibition of PTPMT1 activates TFEB via mobilization of calcineurin catalytic activity (Fig. 4b–e and Supplementary Fig. 4b, c). DG-induced TFEB activation was resistant to calcineurin perturbation at EC90, but was inhibited to some extent at EC50 (Fig. 4b–e and Supplementary Fig. 4c). In contrast, IKA-induced TFEB activation was calcineurin independent (Fig. 4b–e and Supplementary Fig 4c). Consistent with that, AD, but not DG and IKA, induced dose-dependent nuclear translocation of nuclear factor of activated T cells (NFAT)^32^, which is known to depend on the activity of calcineurin (Supplementary Fig. 4d).

An additional primary response to both elevated cytosolic Ca^2+^ and nutrient starvation is activation of AMP-activated protein kinase (AMPK)^33^. AMPK mediates biological responses to caloric restriction through both mTOR-dependent and mTOR-independent mechanisms and is engaged directly by calcium/calmodulin-dependent protein kinase kinase beta (CaMKKβ) upon elevation of cytosolic Ca^2+^. As expected, chemical activation of AMPK either directly with 5-aminoimidazole-4-carboxamide ribonucleotide (AICAR)^34^ or indirectly with metformin^35^ was sufficient to induce TFEB nuclear accumulation (Supplementary Fig. 4e). Dorsomorphin (Compound C), an AMPK inhibitor and STO-609, a CaMKKβ inhibitor^36^, both significantly inhibited TFEB nuclear translocation induced by IKA and AD, but not DG. Furthermore, IKA and AD, but not DG, induced activating phosphorylation of AMPK and its downstream substrate acetyl-CoA carboxylase (ACC) in a dose-dependent manner (Supplementary Fig. 4f, g). Finally, the effects of AD and IKA, but not DG, on TFEB can be reversed by addition of cell-permeable pyruvate, which increases intracellular ATP level and promotes inactivation of AMPK (Fig. 4f, g). These observations indicate that distinct Ca^2+^-dependent mechanisms mediate AD, DG, and IKA induction of TFEB. AD activity is calcinuerin- and AMPK-dependent, IKA is calcinuerin-independent but AMPK-dependent, and DG is relatively independent of both calcinuerin and AMPK.

### Different Ca^2+^ stores contribute to TFEB activation by DG and AD versus IKA

**Fig. 5:**
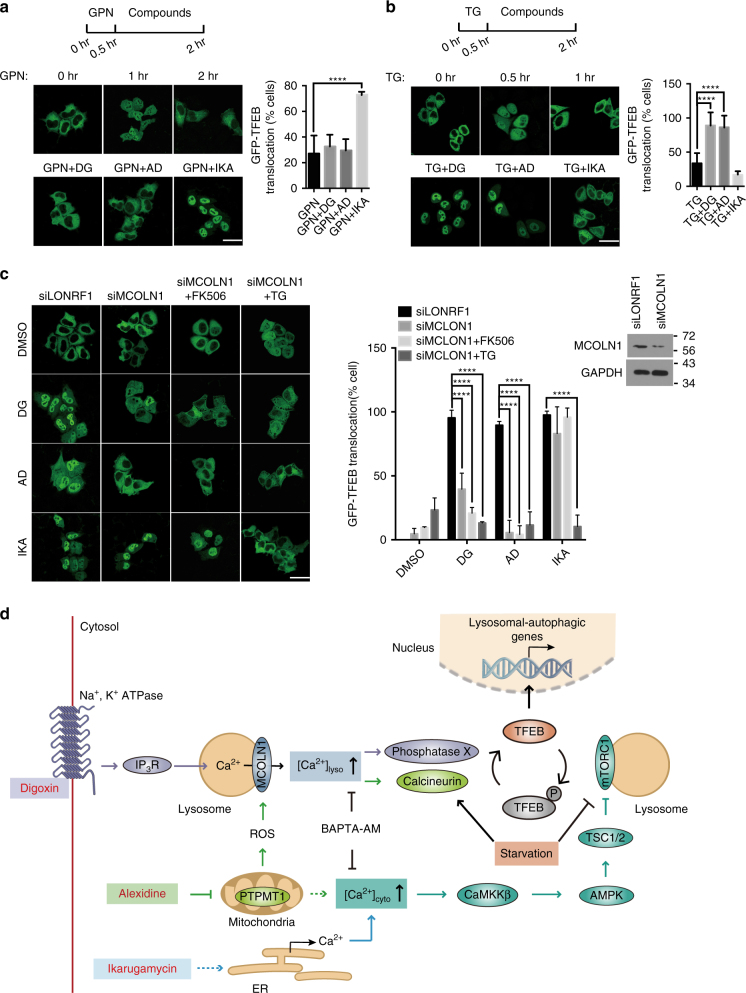
Distinct Ca^2+^-dependence of small-molecular agonists of TFEB. **a**, **b** GFP-TFEB HeLa cells before and after a 30-min treatment of 200 μM GPN (upper panel of **a**) or 300 nM TG (upper panel of **b**) and cells pretreated 30 min with GPN followed by a treatment with DG, AD, and IKA (lower panel). The graph represents the percentage of cells with GFP-TFEB translocation under these conditions (mean ± s.d. for *n* = 3 independent experiments, ****p < 0.0001 by one-way ANOVA). Scale bar, 20 μm. **c** Representative images of GFP-TFEB HeLa cells treated with DG, AD, and IKA together with control siRNA (siLONRF1) treatment, siRNA-mediated inhibition of MCOLN1 (siMCOLN1) or in combination with 5 μM FK506 (4 h) and 300 nM TG (30 min pretreatment). The graph represents the percentage of cells with GFP-TFEB translocation under these conditions (mean ± s.d. for *n* = 3 independent experiments, ****p < 0.0001 by two-way ANOVA). Scale bar, 20 μm. Inset shows the immunoblot of cells treated with siLONRF1 or siMCOLN1. **d** Schematics of proposed mechanism of actions of DG, AD, and IKA. Cardiac glycosides, such as DG, promote binding of their molecular target (Na^+^-K^+^-ATPase) to IP3R. IP3R-dependent ER Ca^2+^ release then recharges lysosomal Ca^2+^ stores through an unclear mechanism, enabling lysosomal Ca^2+^ release through mucolipin-1 (MCOLN1). AD targets PTPMT1 in mitochondria to perturb mitochondrial function and induce ROS release. The lysosomal Ca^2+^ channel mucolipin-1 responds to elevated ROS, which results in a lysosomal Ca^2+^ release. This activates calcineurin and likely additional unknown phosphatases, which de-phosphorylate TFEB and promote nuclear translocation. Furthermore, mTORC1 maintains inhibitory TFEB phosphorylation under nutrient-rich conditions. AD and IKA both increase cytosolic Ca^2+^ levels resulting in CaMKKβ and AMPK pathway activation, which in turn negatively regulates mTORC1 to promote TFEB activation. DG also inhibits the activity of mTORC1 through an unknown mechanism. Activation of TFEB promotes lysosomal biogenesis and autophagy and upregulates genes promoting lipid metabolism. DG-, AD-, and IKA-related proteins/pathways were coded in purple, green, and light blue. Shared proteins/pathways between DG and AD or AD and IKA were coded in cyan and dark blue

Lysosomes, mitochondria and endoplasmic reticuli (ER) are the major compartmentalized Ca^2+^ stores in cells^37^. The seemingly distinct mechanistic links between chemically induced cytosolic Ca^2+^ and TFEB activation lead us to consider discrete Ca^2+^ sources as a potential specificity determinant. Glycyl-l-phenylalanine 2-napthylamide (GPN) is a lysosome-disrupting agent that is commonly used to deplete lysosome-specific Ca^2+^ stores^38^. A 30 min pretreatment of GPN was sufficient to disrupt TFEB nuclear translocation induced by DG and AD, but had almost no effect on IKA-treated cells (Fig. 5a). In contrast, ER-specific depletion of Ca^2+^ by acute treatment with thapsigargin (TG), a specific inhibitor of ER Ca^2+^ ATPase SERCA pump^39^, selectively decreased TFEB nuclear translocation induced by IKA (Fig. 5b). This suggests selective perturbation of lysosomal calcium pools by DG and AD versus ER calcium pools by IKA. Notably, RNAi-mediated depletion of the principal Ca^2+^ channel in lysosomes, mucolipin-1 (MCOLN1), suppressed TFEB nuclear translocation induced by DG and AD, but not IKA (Fig. 5c). MCOLN1 can be activated by ROS in the cells and thus activate TFEB in a lysosomal Ca^2+^/calcineurin-dependent manner^40^. As AD impairs mitochondrial function through targeting PTPMT1, we tested intracellular ROS levels as a potential explanation for the consequences of AD on TFEB activation. Consistent with this hypothesis, tert-Butyl hydroperoxide (TBHP) and AD induced comparable ROS production and TFEB nuclear translocation. Importantly, the effects of both compounds on these phenotypes were abolished by the membrane-permeable antioxidant n-acetyl-cysteine (NAC) (Supplementary Fig. 5a–c). With respect to DG, we noted that cardiac glycosides have been reported to promote the binding of Na^+^-K^+^ ATPase to IP3R, which can in turn induce downstream Ca^2+^ release through IP3R^41^ and refuel lysosomal Ca^2+^ store. A relatively specific IP3R inhibitor Xestosporin C (Xesto), as well as a siRNA-mediated depletion of type 1 IP3R (IP3R1), profoundly attenuated TFEB nuclear translocation induced by IKA, AD, and DG in a time-dependent manner (Supplementary Fig. 5d–i) consistent with the direct engagement of ER- versus lysosome-specific (indirectly through ER) Ca^2+^ by these compounds. Taken together, these observations indicate that distinct calcium stores mediate TFEB activation by DG and AD versus IKA (Fig. 5d).

### TFEB agonists mitigate metabolic syndromes and extend lifespan in vivo

**Fig. 6:**
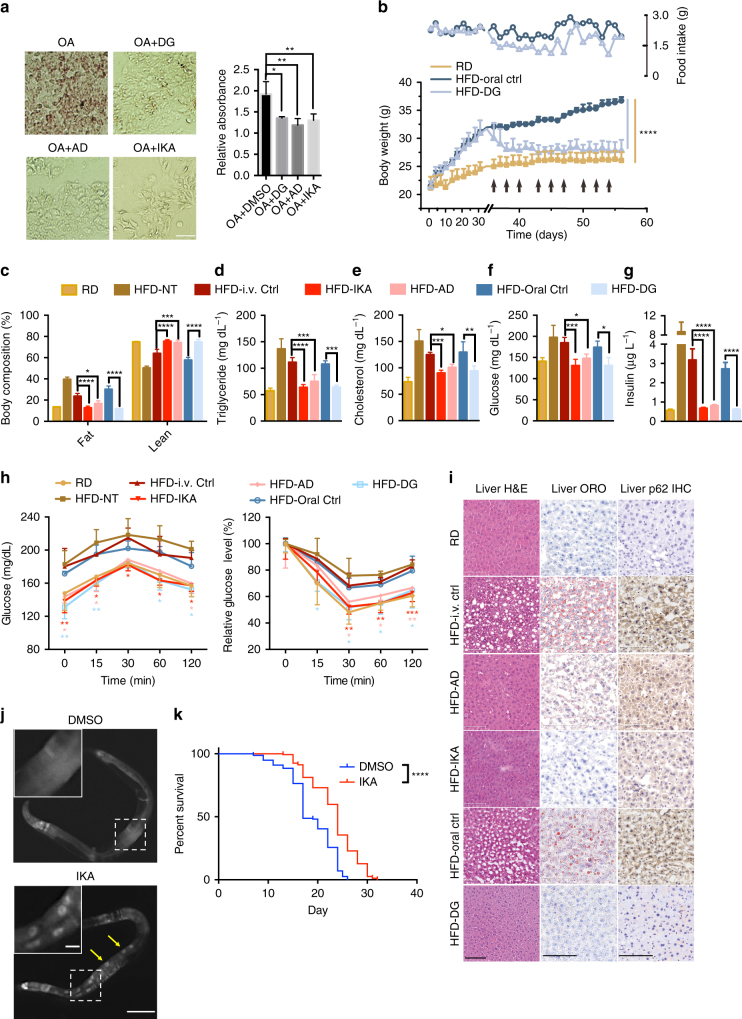
Small-molecule agonists of TFEB promote lipid metabolism and extend lifespan in vivo. **a** Bright-field images showing oil red O (ORO)-stained HepG2 cells treated with 1 mM oleic acid (OA) in combination with 370 nM DG, 3.3 μM AD, and IKA. The graph was obtained by absorbance reading of ORO using plate reader (bars represent mean ± s.d. *p < 0.05, **p < 0.01, ***p < 0.001 by two-way ANOVA). Scale bar, 50 μm. **b** Food uptake (open symbols and right *y*-axis) and body weight change (solid symbols and left *y*-axis) of mice fed with regular diet (RD), high-fat diet with oral injection of DG solvent (HFD-oral ctrl) and HFD with DG oral injection (HFD-DG) three times a week starting from Day 35 as indicated by the arrows (bars represent mean ± s.d. *p < 0.05, **p < 0.01, ***p < 0.001, ****p < 0.0001, HFD-DG compared with HFD-oral ctrl group by using two-way ANOVA). **c** Whole-body composition analysis (EchoMRI) of the same mice as in **b** and Supplementary Fig. 6 **c**, **d** after 3 weeks of treatment with compounds or their corresponding controls. **d**–**g** Total serum triglyceride, cholesterol, glucose, and insulin levels in compound-treated mice or their corresponding control mice after 3 weeks of treatment with compounds or their corresponding controls. **h** Glucose levels at indicated time points after glucose challenge (left panels) and insulin challenge (right panels). In **b**–**h**, *n* = 3–5 mice per group, bars represent mean ± s.d. *p < 0.05, **p < 0.01, ***p < 0.001, ****p < 0.0001 by two-way ANOVA. **i** Hematoxylin and eosin (H&E) staining, ORO staining and immunohistochemistry staining against p62 of liver sections isolated from mice after 3 weeks of treatment with or without compounds. HFD-i.v. ctrl, mice injected with empty PEG-PLA nanoparticles. Scale bars, 100 μm. **j** Representative images of HLH-30::GFP nuclear translocation in *dal-1(dt2300);* sqIs19 [hlh-30p::hlh-30::GFP] *C*. *elegans* treated with 5 μM IKA or DMSO. Insets show enlarged images from the white boxes in the main images. Yellow arrows denote nuclear localized HLH30::GFP. Scale bar, 100 μm and 10 μm (insets). **k** The Kaplan–Meier curves of *dal-1(2300);fem-1(hc17ts)* mutant *C*. *elegans* treated with 5 μM IKA or DMSO (*n* = 2 independent experiments, ****p < 0.0001 by log-rank test)

In animals, TFEB plays a key role in promoting lipid metabolism during starvation, at least in part through global transcriptional activation of peroxisome proliferator-activated receptor γ coactivator 1 α (*Ppargc1α*) and peroxisome proliferator-activated receptor α (*Ppar1α*)^7, 42^. Consistent with physiologically pertinent TFEB activation, DG, AD, and IKA significantly ameliorated oleic acid-induced lipid accumulation in human hepatocytes (Fig. 6a). Importantly, oral administration of DG normalized body weight, body composition and circulating cholesterol, triglycerides, glucose and insulin levels in mice challenged with a high-fat diet (Fig. 6b–g). In contrast, DG did not alter the body weight of the lean mice (Supplementary Fig. 6a). For in vivo analysis of AD and IKA, compounds were encapsulated into biocompatible, biodegradable polyethylene glycol–*b*-poly (d, l-lactic acid) (PEG-PLA) nanoparticles that are liver-trophic^43, 44^ (Supplementary Table 1). Controlled and sustained release of AD and IKA persisted for more than 2 days in an in vitro setting that mimics the in vivo environment (Supplementary Fig. 6b), supporting the 3-times-a-week treatment regimen. Like DG, significant normalization of body weight/composition and blood chemistry was observed upon high-fat challenge relative to control groups (Fig. 6c–g and Supplementary Fig. 6c, d). Moreover, compound-treated mice also displayed improved glucose and insulin tolerance relative to control animals (Fig. 6h). Liver histology revealed amelioration of high-fat diet-induced steatosis, which corresponded to upregulation of *Ppargc1α*, *Ppar1α*, and *Fgf21*^45–47^ by DG, AD and IKA (Fig. 6i and Supplementary Fig. 6e). Compound treatment also reversed p62/SQSTM1 accumulation in hepatocytes, suggesting enhanced autophagic flux in these mice (Fig. 6i). No obvious toxicity to major organs was observed in any treatment group, nor was it observed in the in vitro experiments (Supplementary Fig. 6f, g). An overnight fast in mice is sufficient to induce a transient increase in hepatic lipid accumulation as a consequence of adipose tissue lipolysis^48^, a phenotype that is exacerbated by chloroquine (CQ) (Supplementary Fig. 6h, i). Notably, this effect can be improved by co-administration of DG, consistent with an enhanced endolysosomal function engaged by TFEB in hepatocytes (Supplementary Fig. 6j–l); an effect also consistent with DG-dependent reduction in p62/SQSTM1 accumulation (Supplementary Fig. 6h). These observations indicate that TFEB activation induced by DG, AD, and IKA engages lipid catabolism and can revert physiologically pertinent metabolic syndromes.

Importantly, TFEB has recently been shown to be required for the lifespan extension induced by starvation/calorie restriction and autophagy in vivo^8^. Thus, we investigated if chemically activated TFEB was sufficient to modulate lifespan. The nematode *C*. *elegans* was selected as a relevant animal model, as the *C*. *elegans* TFEB ortholog HLH-30 engages the CLEAR motif to induce the expression of orthologous TFEB targets and autophagy in vivo^7, 8^. A sterile *fem-1 (hc17ts)* and *dal-1 (dt2300)* background was chosen to facilitate the lifespan assay and accumulation of xenobiotics^49^, respectively. *fem-1(−)* animals have temperature-sensitive fertility defects, which simplifies lifespan analysis as mothers do not need to be continuously separated from progeny. *dal-1(−)* worms are healthy but have permissive oral chemical bioavailability via enhanced intestinal absorption. Among the three compounds, IKA-treatment was found to induce nuclear accumulation of GFP-tagged HLH30 within intestinal cells in adults (Fig. 6j). Notably, IKA significantly extended the lifespan of the *fem-1(−);dal-1(−)* animals (Fig. 6k and Supplementary Table 2).

## Discussion

In this study, we leveraged a facile high-throughput phenotypic screening strategy to identify small-molecule TFEB agonists. Mechanism of action analyses of the lead hits has revealed distinct mechanistic mobilization of intracellular calcium stores that can result in productive engagement of TFEB activity in cells. These mechanisms presumably translate, at least in part, to in vivo activity corresponding to amelioration of diet-induced lipid metabolic disorders and lifespan extension.

The conventional target-centric approach for drug discovery, without consideration of optimal cellular mechanisms of action, may result in the current high attrition rates and low productivity in pharmaceutical research and development. Herein, we carefully designed a phenotypic screen for TFEB agonists with therapeutic potential. To avoid lysosomal stressors such as bafilomycin A1 and chloroquine, we first selected for agents that promote autophagic flux in a GFP-labeled LC3 cell model. A broad dynamic activity window was achieved by using UPS nanobuffers that clamp the luminal pH of endocytic organelles and thus arrest them at distinct maturation stages (Fig. 1a). Compared to membrane-permeable small-molecular bases (e.g., chloroquine and NH_4_Cl, Fig. 1b), the UPS collection facilitates high-resolution characterization of luminal pH-specific cell signaling and metabolic processes^23^. UPS_4.4_ was leveraged for these studies because it selectively perturbs lysosomal degradation of macromolecules and maximally preserves signaling functions. Moreover, the UPS_4.4_-dependent arrest of lysosomal pH can be overcome by enhanced lysosomal proton import via v-ATPase activity. The significantly increased signal window by UPS_4.4_ allowed improved accuracy and high-throughput screening of a large collection of compounds. A secondary high-content screen for agents that induce TFEB nuclear translocation allowed the discovery of novel TFEB agonists including several FDA-approved drugs, marine-derived natural product fractions and synthetic small molecules.

Starvation-induced autophagy has been shown to be regulated by lysosomal Ca^2+^-mediated TFEB activation^7^. Here, we find that an orchestrated Ca^2+^ release from distinct organelles (ER, lysosomes, mitochondria) was selectively induced by each of the three distinct TFEB agonists, which indicates Ca^2+^ homeostasis as a paramount biological modulator of TFEB activity. These Ca^2+^ sources selectively engaged either a global Ca^2+^-sensing CaMKKβ-AMPK pathway or a local Ca^2+^-sensing MCOLN1-calcineurin pathway, to release the ‘‘brake’’ (inhibition of mTORC1 through AMPK) and/or push the ‘‘accelerator’’ (activation of TFEB phosphatases) on the TFEB activation cycle (Fig. 5d). We found that DG-induced lysosomal calcium release through MCOLN1, which is likely refueled by ER calcium release from IP3R. Given the direct contact of cardiac-glycoside-bound Na^+^-K^+^ ATPase with the IP3 receptor on ER^50^, we suspect the mode of action of DG is Na^+^-K^+^ ATPase- and IP3R-dependent calcium mobilization and consequent downstream phosphatase activation (Fig. 5d). Surprisingly, DG does not depend on the activity of calcineurin (Fig. 4b–e), suggesting the presence of an uncharacterized Ca^2+^-responsive TFEB phosphatase(s). In contrast, we found that the activation of TFEB by AD is through ROS-dependent activation of the MCOLN1-calcineurin pathway via direct perturbation of the mitochondrial protein PTPMT1 (Fig. 4b–e). Whether or not different MCOLN1 activation mechanisms account for engagement of different TFEB phosphatases now presents as an intriguing opportunity for future investigation. Unlike DG and AD, we found that IKA triggers conventional ER-mediated Ca^2+^-release to activate the CaMKKβ-AMPK pathway (Fig. 4b, c and Fig. 5a–c). Identification of the direct molecular target of IKA will likely shed new light on mechanisms governing organellar communication and selective mobilization of distinct calcium stores.

TFE3 and TFEB regulate overlapping gene sets and have similar biological effects overexpressed^18^. Furthermore, both are regulated by mTORC1-dependent phosphorylation on lysosomal surfaces and by phosphatase calcineurin^10, 17–19^. Given the overlapping mechanistic profiles of TFE3 and TFEB it is likely that the compounds described here can activate both TFEB and TFE3. A recent study examining the physiological roles of TFE3 versus TFEB found partial redundancy in several tissues^20^. However, under conditions such as starvation, high-fat diet and exercise, TFE3 and TFEB are both required and their roles are cooperative. Therefore, the beneficial effects of the compounds we observed in HFD-fed mice are likely a consequence of the combined inhibition of both TFEB and TFE3.

Current lipid-lowering drugs are more targeted toward inhibiting cholesterol synthesis or reabsorption, such as HMG-CoA (3-hydroxy-3-methylglutaryl-coenzyme A) reductase inhibitors^51^, bile-acid binding resins^52^, and cholesterol absorption inhibitors^53^. As the role of lysosomes and autophagy in macromolecule metabolism becomes clearer, boosting this innate cellular clearance machinery, such as the TFEB agonists, offers a new therapeutic strategy for treatment of these diseases. In fact, pharmacological intervention of targets upstream of the TFEB-autophagy pathway, such as mTORC1 inhibitors^54, 55^ and AMPK activators^56, 57^, have already been shown to hold considerable promise to reverse metabolic abnormalities and even to extend lifespan. Genetic studies have already demonstrated the role that TFEB plays under these pathophysiological conditions. The newly discovered small-molecule TFEB agonists will facilitate studies focused on new TFEB biology and will promote viable pharmacological strategies to tackle metabolic syndromes, ageing and age-related diseases.

## Methods

### Chemical reagents

The TMR-NHS, Cy5-NHS, BODIPY-NHS, and Cy3.5-NHS esters were purchased from Lumiprobe Corp. (FL, USA). 2-Aminoethyl methacrylate (AMA) was purchased from Polyscience Company. Monomers 2-(dibutylamino) ethyl methacrylate (DBA-MA) and 2-(dipentylamino) ethyl methacrylate (D5A-MA) were prepared according to the method described in our previous work, as well as the PEO macroinitiator (MeO-PEO_114_-Br). N,N,N′,N″,N″’-Pentamethyldiethylenetriamine (PMDETA) and poly(ethylene glycol)-*b*-poly(d,l-lactide) (PEG-PLA, *M*_n_ ~ 5,000 Da for each segment) were purchased from Sigma-Aldrich. (2-Hydroxypropyl)-β-cyclodextrin (HPβCD) was purchased from Fisher Scientific Inc. Amicon ultra-15 centrifugal filter tubes (MWCO = 100 K) were obtained from Millipore (MA). Other reagents and organic solvents were analytical grade from Sigma-Aldrich or Fisher Scientific Inc. Digoxin, proscillaridin A, alexdine dihydrochloride, ikarugamycin, bafilomycin A1, FK506, cyclosporine A, dorsomorphin (compound C), AICAR, metformin, STO-609, thapsigarigin, N,N,N′,N′-Tetrakis(2-pyridylmethyl)ethylenediamine (TPEN), and oleic acid were purchased from Sigma-Aldrich; Torin 1 was from Tocris Bioscience; Xestosporin C was from Cayman Chemical; BAPTA-AM, Fura-2-AM, calcium calibration buffer kit (with 0 and 10 mM EGTA), Hoechst 33342, CellROX green, tert-Butyl hydroperoxide (TBHP) and NAC were from Invitrogen; Gly-Phe β-naphthylamide (GPN) from Santa Cruz Biotechnology; Magic Red^TM^-(RR)_2_Cathepsin B assay kit from Marker Gene Technologies, Inc.

### Antibodies and immunoblots

The following antibodies were used for immunoblot: GAPDH (cat. 5174, 1:1,000), SQSTM1/p62 (cat. 5114, 1:1,000), TFEB (cat. 4240, 1:1,000), LAMIN A/C (cat. 2023, 1:1000), AMPKα-pThr172 (cat. 2535, 1:1,000), total AMPKα (cat. 5831, 1:1,000), ACC-pS79 (cat. 1:1000), total ACC (cat. 3676, 1:1,000), S6-pS235/236 (cat. 4858, 1:1,000), total S6 (cat. 2217, 1:1,000), Na^+^, K^+^ -ATPase α1 (cat. 3010, 1:1,000), p70-S6K-pThr389 (cat. 9234, 1:1,000), total p70-S6K (cat. 2708, 1:1,000), TSC2-pThr1462 (cat. 3611, 1:1,000), total TSC2 (cat. 3635, 1:1,000), AKT-pThr308 (cat. 2965, 1:1,000), pan AKT (cat. 4691, 1:1,000), IP3R1 (cat. 3763, 1:1,000), and TFE3 (cat. 14779, 1:1000) were from Cell Signaling Technology and PPP3CB (cat. ab191374, 1:1000) was from Abcam; the following antibody was used for immunofluorescence: TFEB (cat. sc-48784, 1:100) from Santa Cruz Biotechnology and NFAT1 (cat. 5861, 1:100) and LAMP1 (cat. 9091, 1:200) from Cell Signaling Technology; the following antibody was used for immunohistochemistry: p62/SQSTM1 (cat. ab91526, 1:200) from Abcam. Cells were collected in 2× sample buffer, boiled, and sonicated for immunoblot analysis using standard western blot protocols. Uncropped blots were shown in Supplementary Fig. 7.

### Cell culture and siRNA transfection

HeLa cells and MEFs were purchased from ATCC and were cultured in Dulbecco's modified Eagle's medium (DMEM, Invitrogen) with 10% fetal bovine serum and 1% antibiotics (Invitrogen). Earle’s Balanced Salt Solution (EBSS, 10×, Sigma) was diluted to 1x with Milli-Q water supplemented with 2.2 g L^−1^ sodium bicarbonate (Sigma). HeLa cells that stably express GFP-TFEB and GFP-LC3, and *p53*^*−/−*^ and *p53*^*−/−*^ and *TSC2*^*−/−*^ MEFs and HepG2 cells were generous gifts from Dr. Shawn Ferguson (Yale University, USA), Dr. Beth Levine (UT Southwestern Medical Center, USA), Dr. James Brugarolas and Dr. Yihong Wan (UT Southwestern Medical Center, USA), and were cultured under the same conditions as described above. In the GFP-LC3 chemical screen, 2 mM NH_4_Cl (Sigma) was supplemented in DMEM. All cell-based studies were performed with 25 mM HEPES buffer in a humidified chamber with 5% CO_2_. All cell lines have been tested for mycoplasma contamination using a MycoFluor^TM^ Mycoplasma Detection Kit (Invitrogen). RNAi was performed by transfecting siRNA oligos (Dharmacon, Inc.) via reverse transfection using RNAiMax (Life Technologies) according to the manufacturer’s instructions. A pool of four siRNA oligos targeting each gene was used to dilute off-target effects. Pools of four siRNAs targeting LONRF1 were used for transfection controls.

### Synthesis of PEO-*b*-PR block copolymers

In a typical procedure using PEO-*b*-PDBA_80_ (UPS_5.3_) as an example, DBA-MA (1.92 g, 8 mmol), PMDETA (21 μL, 0.1 mmol) and MeO-PEO_114_-Br (0.5 g, 0.1 mmol) were charged into a polymerization tube. The monomer and initiator were dissolved in a mixture of 2-propanol (2 mL) and dimethylformamide (2 mL). Three cycles of freeze–pump–thaw were performed to remove the oxygen, then CuBr (14 mg, 0.1 mmol) was added into the tube protected by nitrogen, and the tube was sealed in vacuo. After 8 h polymerization at 40 °C, the reaction mixture was diluted in 10 mL tetrahydrofuran (THF), and the mixture was passed through a neutral Al_2_O_3_ column to remove the catalyst. The organic solvent was removed by rotovap. The residue was dialyzed in distilled water and lyophilized to obtain a white powder.

### Preparation of UPS nanoparticle solutions

In a typical procedure, 10 mg UPS polymer was dissolved in 500 μL THF (UPS_4.4_) or methanol (always-on/OFF-ON UPS_5.3_). For always-on/OFF-ON UPS_5.3_ nanoprobes, BODIPY-conjugated polymer, and Cy3.5-conjugated polymer was mixed with a 3:2 weight ratio. The solution was added to 10 mL Milli-Q water drop by drop. Four to five filtrations through a micro-ultrafiltration system (<100 kDa, Amicon Ultra filter units, Millipore) were used to remove the organic solvent. The aqueous solution of UPS nanoparticles was sterilized with a 0.22 μm filter unit (Millex-GP syringe filter unit, Millipore).

### High-throughput GFP-LC3 chemical screen

GFP-LC3 HeLa cells were seeded in 384-well plates. UPS_4.4_ nanobuffer solutions were added the following day, and compounds (2.5 μM) were added for 4 h on the third day. UPS_4.4_–only cells were used as positive controls and wild-type HeLa cells were used as negative controls. Cells were then fixed with 4% formaldehyde, stained with 0.01% Hoechst 33324, and then sealed and read on with PHERAstar *FS* HTS microplate reader (BMG LABTECH). A saline-only plate was used to control background signals. Genedata Screener software (GeneData, Inc. Basel, Switzerland) was used to process and analyze the results. For each plate, the raw fluorescence GFP values were normalized with corresponding Hoechst signals after background-correction for all wells. The converted data was then normalized using Equation 1. Normalized well values were then corrected for position artifacts based on GeneData proprietary pattern detection algorithms. Finally, robust *Z*-scores were calculated using Equation 2.

1 $${\rm Normalized\,data} = {\textstyle{{{\rm Converted\,data}_{\rm {sample}} - {\rm Median\,of\,converted}\, {\rm data}_{{\rm DMSO}}} \over {{\rm |Median\,of\,converted\,data}_{{\rm positive\,control}} - {\rm Median\,of\,converted\,data}_{{\rm DMSO}}|}}} \times 100.$$

2 $${\rm Robust}\,Z\,{\rm score} = \frac{{{\rm Converted\,data}_{{\rm sample}} - {\rm Median\,of\,converted\,data}_{{\rm all\,sample}}}}{{{\rm Converted\,robust\,standard\,deviation}_{{\rm all\,sample}}}}$$

For the primary screen, each compound was tested as *N* = 1, and primary hits were selected with robust *Z*-scores less than −3. For the validation screen, the primary hits were assayed in triplicate. For each compound, the normalized activity values were condensed to a single value (condensed activity score) using the “Robust Condensing” method in Genedata Screener. The condensed activity is the most representative single value of the triplicates. Thirty compounds with lowest condensed activity values and robust *Z*-score values were selected as the final hits.

### High-content GFP-TFEB chemical screen

GFP-TFEB HeLa cells were seeded in 384-well plates. Compounds were added for 4 h the following day. Bafilomycin A1 (250 nM) treated cells were used as positive controls and dimethyl sulfoxide (DMSO)-treated cells as negative controls. Cells were fixed with 4% formaldehyde, stained with 0.01% Hoechst 33324, and then imaged on a GE IN Cell 6000 automated microscope with a 10X objective. Images were collected using 405 and 488 nm laser lines with DAPI (4',6-diamidino-2-phenylindole) and FITC (fluorescein isothiocyanate) emission filters. Images were analyzed using the GE IN Cell Analyzer Workstation software. Briefly, nuclei were segmented using the Hoechst channel and the cytoplasm was segmented using the GFP channel. For each cell, the mean GFP intensity in each compartment was measured and used to calculate the nuclear to cytoplasmic (N/C) TFEB-GFP ratio. The same method as mentioned above was used to generate the top 30 compounds with highest condensed activity values and robust *Z*-score values.

### Isolation and purification of ikarugamycin

SW201073 was extracted from marine-derived bacterium strain SNB-040 isolated from a sediment sample collected from Sweetings Cay, Bahamas. Bacterial spores were collected via a stepwise centrifugation as follows: 2 g of sediment was dried over 24 h in an incubator at 35 °C and the resulting sediment added to 10 mL sH_2_O containing 0.05% Tween 20. After vigorous vortex for 10 min, the sediment was centrifuged at 18,000 rpm for 25 min (4 °C) and the resulting spore pellet collected. The resuspended spore pellet (4 mL sH_2_O) was plated on an acidified JMA media, giving rise to individual colonies of SNB-040 after 2 weeks. Analysis of the 16S rRNA sequence of SNB-040 revealed 99% identity to *Streptomyces phaeochromigenes*.

Bacterium SNB-040 was cultured in 20 × 2.8 L Fernbach flasks each containing 1 L of seawater-based medium (10 g starch, 4 g yeast extract, 2 g peptone, 1 g CaCO_3_, 40 mg Fe_2_(SO_4_)_3_•4H_2_O, 100 mg KBr) and shaken at 200 rpm at 27 °C. After 7 days of cultivation, sterilized XAD-7-HP resin (20 g L^−1^) was added to absorb the organic products, and the culture and resin were shaken at 200 rpm for 2 h. The resin was filtered through cheesecloth, washed with deionized water, and eluted with acetone. The acetone-soluble fraction was dried in vacuo to yield 4.5 g of extract.

Crude extract of SNB-040 was fractionated using reverse phase flash column chromatography (C18) with a stepwise gradient (20–100%) MeOH/H_2_O. Fractions were analyzed by Liquid chromatography–mass spectrometry (LC–MS) using an analytical C18 column and gradient from 10–100% acetonitrile/water (0.1% formic acid) over 17 min (0.7 mL min^−1^), followed by 100% acetonitrile for 5 min. Ikarugamycin elutes at 21 min on this LC–MS method. Fractions containing ikarugamycin were combined, dried, and purified using reverse phase HPLC (phenyl-hexyl column, Phenomenex Luna, 250 × 10.0 mm, 5 μm) at 80% acetonitrile/water (0.1% formic acid) and ikarugamycin *t*_R_ = 12.5 min with a strong ultraviolet (UV) absorbance at 254 nm. Ultimately a white amorphous solid (ikarugamycin) with a *λ*_max_ absorption of 250 and 325 nm with *m*/*z* [M + H] of 479.2 was purified. ^1^H NMR (600 MHz, DMSO-d6) *δ*: 7.69 (dd, *J* = 5.7 Hz, 1H), 7.46 (d, *J* = 15.3 Hz, 1H), 6.45 (br s, 1H), 6.05 (dd, *J*_1_ = 15.3 Hz, *J*_2_ = 9.9 Hz, 1H), 5.94 (dt, *J*_1_ = 15.5 Hz, *J*_2_ = 14.4 Hz, 1H), 5.86 (d, *J* = 9.6 Hz, 1H), 5.82 (dd, *J*_1_ = 14.4 Hz, *J*_2_ = 2.0 Hz, 1H), 5.72 (dd, *J*_1_ = 9.6 Hz, *J*_2_ = 2.0 Hz, 1H), 3.38 (m, 1H), 3.30 (m, 1H), 3.28 (m, 1H), 2.49 (m, 1H), 2.39 (m, 1H), 2.25 (m, 2H), 2.10-2.09 (m, 2H), 2.02 (m, 1H), 1.99 (m, 1H), 1.71 (m, 1H), 1.63 (m, 1H), 1.53 (m, 1H), 1.45 (m, 1H), 1.33-1.31 (m, 3H), 1.26 (m, 1H), 1.16-1.09 (m, 3H), 0.91 (dd, *J*_1_ = 8.3 Hz, *J*_2_ = 7.2 Hz, 3H), 0.86 (d, *J* = 7.2 Hz, 2H), 0.66 (m, 1H). ^13^C NMR (100 MHz, DMSO-d6) *δ*: 195.3, 181.3, 176.6, 165.6, 140.1, 137.8, 132.0, 130.2, 129.1, 125.0, 101.9, 58.4, 48.6, 48.3, 47.3, 46.7, 46.5, 42.1, 41.1, 38.3, 37.9, 36.9, 33.7, 27.2, 24.8, 21.6, 21.1, 17.7, 13.1.

### Dose–response assays

Wild-type, GFP-LC3 and GFP-TFEB HeLa cells were seeded in 96-well plates and treated with half log dilutions of compounds in triplicates. Treatment, data acquisition and analysis was identical to that described for the HTS chemical screens. Cathepsin B activity was measured using the Magic Red^TM^-(RR)_2_ Cathepsin B assay kit following a 4 h treatment with compounds at indicated doses. Raw data was background-corrected, log-transformed and fit with the dose–response function with Graphpad Prism (v6.0) software.

### Confocal imaging

All confocal imaging was performed on a Zeiss LSM 700 laser scanning confocal microscope using a 40X/1.3DIC objective. Cells were plated on 4- or 8-well Nunc^TM^ Lab-Tek^TM^ II Chambered Coverglass (Thermo Scientific) and allowed to grow for 24 h. After treatment, cells were fixed in 4% formaldehyde and imaged at excitation wavelengths of 488 nm (GFP, LysoSensor Green or BODIPY) and 560 nm (Cy3.5). ImageJ software (NIH) was used to process and analyze the images.

### Endosomal maturation rates

HeLa cells were plated on 4- or 8-well Nunc^TM^ Lab-Tek^TM^ II Chambered Coverglass and allowed to grow for 24 h. After 4 h treatment with compounds or DMSO, cells were incubated with always-ON/OFF-ON UPS_5.3_ nanoprobes for 5 min in serum-free medium, then washed three times with PBS before imaging. The FI_OFF-ON (BODIPY)_/FI_Always-ON (Cy3.5)_ ratio was quantitated with ImageJ. For each cell, a region of interest was defined as the punctae in cytosol that emitted fluorescent signals from both BODIPY and Cy3.5 channels. Fluorescent intensity ratio was calculated for each intracellular punctate as *R* = (*F*_1_ -* B*_1_)/(*F*_2 _- *B*_2_) where *F*_1_ and *F*_2_ are the fluorescence intensities from BODIPY and Cy3.5 channels respectively, and *B*_1_ and *B*_2_ are the corresponding background values determined from a region on the same images that was near the punctae in the cytosol. All the ratios of each nanoprobe were normalized to their end time-point ratio, and the curves were fit with Graphpad Prism (v6.0) software.

### Fura-2 Ca^2+^ imaging

HeLa cells treated with compounds or DMSO for 4 h were loaded with Fura-2-AM (3 μM) in cell culture medium for 60 min at 37 °C. Cells were washed twice then incubated in fresh medium for 30 min to allow complete de-esterification of intracellular AM esters. Imaging was performed at 37 °C with 5% CO_2_ on an epifluorescent microscope (Deltavision, Applied Precision) equipped with a digital monochrome Coolsnap HQ2 camera (Roper Scientific, Tucson, AZ). Fluorescence images were collected using SoftWoRx v3.4.5 (Universal Imaging, Downingtown, PA). Data were recorded at excitation/emission wavelengths of 340/510 nm (Fura-340 filter) and 387/510 nm (Fura-380 filter). The single band pass excitation filter for Fura-340 and Fura-380 is 26 and 11 nm, respectively, and the band pass of emission filters for Fura-340 and Fura-380 is 84 nm. Intracellular calibration of Fura-2 was accomplished by manipulating the Ca^2+^ levels inside cells using the ionphore ionomycin (20 μM, Sigma-Aldrich) and by incubating cells in buffers with various Ca^2+^ concentrations (calcium calibration buffer kit- Invitrogen). Intracellular fluorescence ratios were determined using ImageJ software. Images were background-corrected by subtracting the mean pixel values of a cell-free region near the region of interest. Fluorescent intensity ratio *R* = *F*_340_/*F*_380_, and Ca^2+^ concentration can be calculated from Equation 3, where *K*_d_ can be obtained from intracellular calibration:

3 $$\left[ {\rm Ca^{2 + }} \right] = K_{\rm{d}} \times \frac{{F_{380,\,{\rm min}}}}{{F_{380,\,{\rm max}}}} \times \frac{{(R - R_{{\rm min}})}}{{(R_{{\rm max}} - R)}}$$

### GFP-TFEB nuclear translocation

GFP-TFEB HeLa cells treated with compounds or DMSO were fixed and stained with Hoechst. Images from at least three different fields per sample were acquired using a 40X objective on a Zeiss confocal microscope and analyzed with ImageJ. In all, 20–30 cells were evaluated from each image for each sample, and three independent experiments were performed to generate the graphed values.

### RNA extraction and qRT-PCR

Total RNA was isolated from MEFs or mouse tissues using RNeasy minipreps (QIAGEN). Complementary DNA (cDNA) was synthesized with the High-capacity RNA-to-cDNA kit (Applied biosystems), and qRT-PCR was performed using TaqMan® Gene Expression Assays (Applied biosystems) for the indicated genes on the LightCycler System (Roche Applied Science). *Gapdh* was used to normalize RNA input. The mouse probes used in this study were: *Tfeb* (Mm00448968_m1), *Ctsa* (Mm00447197_m1), *Mcoln 1* (Mm00522550_m1), *Pparα* (Mm00440939_m1), *Ppargc1α* (Mm01208835_m1), *Fgf21* (Mm00840165_g1), and *Gapdh* (Mm99999915_g1), and the human probes used were *TFEB* (Hs00292981_m1), *CTSA* (Hs00264902_m1), *MCOLN1* (Hs01100653_m1), *PPARGC1A* (Hs00173304_m1), *PPARA* (Hs00947536_m1), *UVRAG* (Hs01075434_m1), *GAPDH* (Hs02758991_g1), and *p62/SQSTM1* (Hs01061917_g1). All probes were from Thermo Fisher Scientific Inc.

### RNA interference (RNAi)

Transfection of siRNA duplexes was used to silence indicated genes. In brief, cells grown in six-well plates were transfected with Lipofectamine® RNAiMAX transfection reagent (Thermo Fisher Scientific) and 100 nM siRNA duplexes targeted against Na^+^-K^+^-ATPase α_1_ subunit (MQ-006111-02), PTPMT1 (MQ-029988-02), PPP3CB (MQ-009704-01), MCOLN1 (MQ-006281-00), TFEB (MQ-009798-02), and TFE3 (MQ-009363-03, Dharmacon). Treated cells were analyzed 48–72 h after transfection.

### Nanoparticle formulation

AD or IKA (1 mg) together with PEG-PLA polymer (9 mg) were first dissolved in 1 mL methanol. The solution was added drop-wise to 10 mL Milli-Q water. Four to five filtrations through a micro-ultrafiltration system (<100 kDa, Amicon Ultra filter units, Millipore) were used to remove the organic solvent and unencapsulated free drugs. The aqueous solution of UPS nanoparticles was sterilized with a 0.22 μm filter unit (Millex-GP syringe filter unit, Millipore). Micelle solutions were then lyophilized and the resulting freeze-dried powder was weighed, dissolved in a mixture of methanol and deionized water (*v*/*v* = 9/1), and analyzed using a Shimadzu UV-1800 UV–Vis spectrophotometer (*λ* = 240 nm, extinction coefficient = 2.0 × 10^4^ M^−1^ cm^−1^) to calculate the total amount of micelle encapsulated drug. Nanoparticles were also characterized by dynamic light scattering to evaluate particle size.

### Mouse model for in vivo compound delivery

All mouse experiments were approved and carried out following the ethical guidelines established by the Institutional Animal Care and Use Committee at UT Southwestern Medical Center. The investigators were not blinded to allocation during experiments and outcome assessment. Four to 6-weeks-old male C57BL/6 J mice were randomly divided into groups fed a regular diet (Harlan Teklad) or a high-fat diet (HFD) containing 60% fat (Research Diets). After 1 month, the HFD mice were grouped so that the average body weights of mice in each group were similar. The grouped mice were orally administered DG (2.5 mg kg^−1^) or its solvent 50% HPβCD solution, or intravenously injected with AD (1 mg kg^−1^) or IKA (0.5 mg kg^−1^) encapsulated in the PEG-PLA nanoparticles or empty nanoparticles, three times a week for three weeks. Body weight and food intake were measured twice a week before treatment and every day after treatment in the middle of the light period. The cage tops containing food pellets were weighed, as well as the spilled food in the bottom of the cage. The food intake was corrected for spillage.

### In vitro compound release from PEG-PLA micelle

AD and IKA release from PEG_5000_-PLA_5000_ micelle was measured using a dialysis method. In a typical procedure, AD or IKA micelle solution (0.5 mL, 10 mg mL^−1^) was added to the upper chamber of a 15 mL mini dialysis tube (3.5 k molecular weight cutoff, Fisher Scientific Inc.) with 1x PBS with 1% Tween 80 (Sigma-Aldrich). At different time points, 1 mL solution was removed from the tube and replaced with 1 mL 1x PBS with 1% Tween 80. The released AD or IKA was determined by measuring the UV–Vis absorbance of the obtained solution based on the standard curves of AD and IKA. Percentage of compound release was plotted as a function of time to show the release kinetics.

### Body composition analysis

At the end of the treatment, the body composition of each mouse was analyzed by EchoMRI (Echo Medical Systems LLC) according to the manufacturer’s instructions.

### Glucose and insulin tolerance tests

For glucose tolerance tests, the mice were orally administered 1 mg g^−1^ glucose (Sigma-Aldrich) after a 4 h fast. For insulin tolerance tests, the mice were intraperitoneally injected with 0.75 milliunit g^−1^ insulin (Humulin R, Eli Lily) after a 4 h fast. Blood was drawn from tail veins at indicated time points after injection. Experiments were performed during light period. Serum glucose levels were analyzed using commercial glucose reagents (Sigma).

### Serum chemistry analysis

At the end of the treatment, blood was collected from the orbital plexus under anesthesia. Serum was frozen in aliquots and stored at −20 °C for further analysis. Specific enzyme kits were used to detect serum levels of triglyceride (Fisher Scientific), cholesterol (Fisher Scientific) and glucose (Sigma-Aldrich).

### Histology

Livers and other organs were dissected and embedded in OCT. Cryostat sections were cut at 10 μm. The sections were stored at −80 °C and subjected to hematoxylin/eosin and oil red O staining following standard protocol. The immunohistochemistry (IHC) staining of p62 was performed following the protocol of Cell Signaling Technology. The primary antibody was from Abcam, and the SignalStain® boost IHC detection reagent and DAB substrate kit were from Cell Signaling Technology. All the sections were imaged using a NanoZoomer 2.0-HT Digital slide scanner (Hamamatzu) and processed using NDP viewer software.

### Cytotoxicity

HeLa cells were plated in a 96-well plate with a white wall and a clear bottom. After 24 h, cells were treated with various doses of DG, AD, and IKA for 4 h. Cells were then washed with PBS three times, and viability was determined immediately or after 72 h using CellTiter-Glo® Luminescent Cell Viability Assay (Promega).

### HLH-30::GFP nuclear localization assay

Adult TX1941 *dal-1(dt2300); sqIs19 [hlh-30p::hlh-30::GFP rol-6(+)]* worms were placed on nematode growth medium (NGM) plates with either a test chemical or 5% DMSO. GFP was scored at various intervals on live worms without mounting using a Zeiss Axio Zoom. V16 fluorescence dissecting microscope equipped with Axiocam 503. No difference was observed between 2 h and overnight treatment.

### *C*. *elegans* lifespan analysis

The *C*. *elegans* mutant strain, *fem-1(hc17ts) IV; dal-1(dt2300)*, obtained from the Caenorhabditis Genetics Center at the University of Minnesota, was cultured on NGM plates seeded with the *E*. *coli* strain OP50 and consistently maintained at 15 °C. For lifespan assays, a total of 12 age-synchronized nematode adults were transferred to eight replicate NGM plates and grown at 25 °C to ensure sterility. Age synchronization was achieved through standard hypochlorite treatments. Eggs were placed on NGM plates supplemented with Streptomycin (100 μg mL^−1^) and seeded with the *E*. *coli* strain, OP50. For compound testing, 200 μL of 10 μM Ikarugamycin in 5% DMSO was spotted directly onto OP50 seeded NGM plates. Control plates were prepared by spotting 200 μL of 5% DMSO directly onto OP50 seeded NGM plates. For the first 10 days of adulthood, *C*. *elegans* were scored once a day as dead or alive by touch stimulation with a platinum wire. After day 10, animals were scored every other day. Nematodes that crawling off the agar plates were censored from subsequent lifespan analysis. Kaplan–Meier statistical analysis was performed using Prism 7 software. Lifespan experiments were done on two separate occasions.

### Statistics

Sample sizes and reproducibility for each figure are denoted in the figure legends. Data were presented as the mean ± s.d. unless specified. Analysis of variance (ANOVA) approaches were used for comparisons among experimental groups that met the normality distribution assumption. If not, the data was log-transformed or a non-parametric *t*-test was used. One-way ANOVA and two-way ANOVA were used for comparison within groups with single or two variables.

### Data availability

The data that support the findings of this study are contained in the figures and the Supplementary Information of this article or are available from the corresponding author upon reasonable request.

## Electronic supplementary material

Supplementary Information
