# Author Correction: Small-molecule TFEB pathway agonists that ameliorate metabolic syndrome in mice and extend *C. elegans* lifespan

**DOI:** 10.1038/s41467-018-04519-8

**Published:** 2018-05-21

**Authors:** Chensu Wang, Hanspeter Niederstrasser, Peter M. Douglas, Rueyling Lin, Juan Jaramillo, Yang Li, Nathaniel W. Oswald, Anwu Zhou, Elizabeth A. McMillan, Saurabh Mendiratta, Zhaohui Wang, Tian Zhao, Zhiqaing Lin, Min Luo, Gang Huang, Rolf A. Brekken, Bruce A. Posner, John B. MacMillan, Jinming Gao, Michael A. White

**Affiliations:** 1 Department of Pharmacology, Simmons Comprehensive Cancer Center, University of Texas Southwestern Medical Center, 5323 Harry Hines Boulevard, 75390 Dallas, TX USA; 2 Department of Cell Biology, University of Texas Southwestern Medical Center, 5323 Harry Hines Boulevard, 75390 Dallas, TX USA; 3 Department of Biochemistry, University of Texas Southwestern Medical Center, 5323 Harry Hines Boulevard, 75390 Dallas, TX USA; 4 Department of Molecular Biology, University of Texas Southwestern Medical Center, 5323 Harry Hines Boulevard, 75390 Dallas, TX USA; 5 Department of Surgery, University of Texas Southwestern Medical Center, 5323 Harry Hines Boulevard, 75390 Dallas, TX USA

Correction to: *Nature Communications* 10.1038/s41467-017-02332-3, published online on 22 December 2017.

The originally published version of this Article contained an error in the spelling of the author Nathaniel W. Oswald, which was incorrectly given as Nathaniel W. Olswald. This has now been corrected in both the PDF and HTML versions of the Article.

