# Supplementary material for: Small-molecule TFEB pathway agonists that ameliorate metabolic syndrome in mice and extend C. elegans lifespan
Source: Nat Commun. 2017 Dec 22;8:2270. doi: 10.1038/s41467-017-02332-3 (PMC5741634; doi:10.1038/s41467-017-02332-3)
Supplement: Supplementary file 1 — Supplementary Information [file 41467_2017_2332_MOESM1_ESM.pdf]

Supplementary Information

Supplementary Figure 1

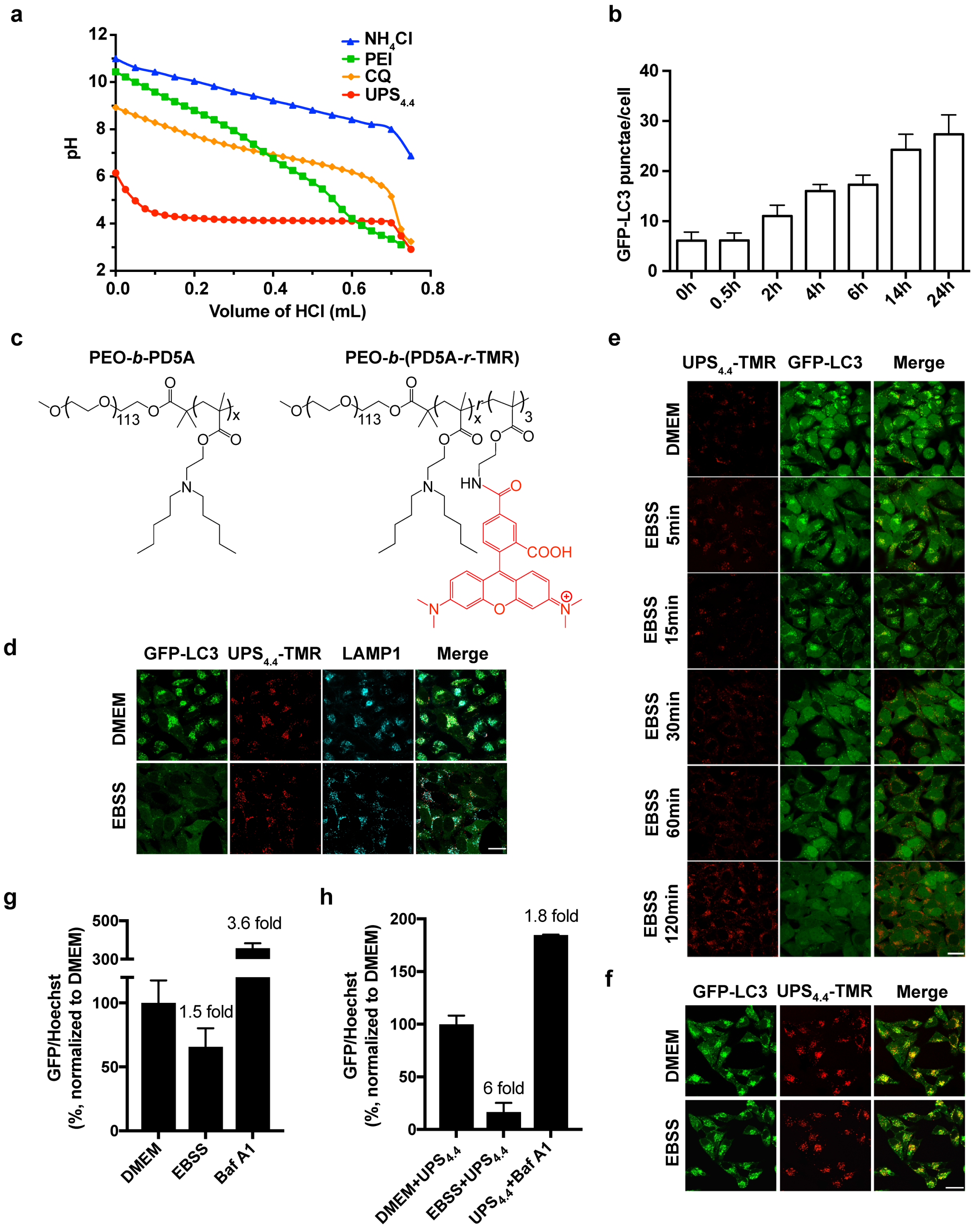

Supplementary Figure 1. A UPS-enabled autophagy assay that broadens dynamic activity window. **(a)** pH titration of solutions containing $UPS_{4.4}$ nanoparticles using 0.4 M HCl. Chloroquine (CQ, pK_a = 8.3 and 10.4) and NH_4Cl, two small molecular bases, and polyethyleneimines (PEI) were included for comparison. **(b)** Number of GFP-LC3 puncta per cell counted after various time of incubation with $UPS_{4.4}$ nanoprobes (n = 20-30 cells from 3 independent experiments). **(c)** Chemical structure of $UPS_{4.4}$ polymers (poly(ethylene oxide)-*b*-poly(2- (dipentylamino)ethyl methacrylate), PEO-*b*-PD5A) with or without tetramethylrhodamine (TMR) conjugation. **(d)** Immunofluorescent images of GFP-LC3 HeLa cells pretreated with $UPS_{4.4}$ labeled with a fluorescent dye TMR ($UPS_{4.4}$-TMR) for 18 hours in DMEM (upper panel) or followed by EBSS treatment for 0.5 hours. LAMP1 was used as a lysosomal marker. Scale bar = 20 μm. **(e)** Time-course images showing the effect of $UPS_{4.4}$-TMR treatment on GFP-LC3 puncta accumulation and clearance in Dulbecco's Modified Eagle Medium (DMEM) and a subsequent nutrient-starvation in Earle's Balanced Salt Solution (EBSS) for indicated time. Scale bar = 20 μm. **(f)** Confocal fluorescent images of GFP-LC3 HeLa cells pretreated with $UPS_{4.4}$-TMR for 18 hours before being treated 100nM baf A1 in DMEM or EBSS for 4 hours. Scale bar = 20 μm. **(g, h)** GFP-LC3 HeLa cells were seeded on a 384-well plate in DMEM, and were treated with (**h**) or without (**g**) $UPS_{4.4}$ for for 18 hours before they were transferred into EBSS or DMEM with 100nM baf A1. Cells that stayed in the original DMEM with $UPS_{4.4}$ being washed off (if applicable) were used as a control. GFP and Hoechst fluorescence were read from a plate reader. GFP/Hoechst ratio was calculated after subtraction of saline background. The fold change of the GFP/Hoechst signal was calculated against the DMEM control group (mean ± s.d. for n = 3 independent experiments).

Supplementary Figure 2

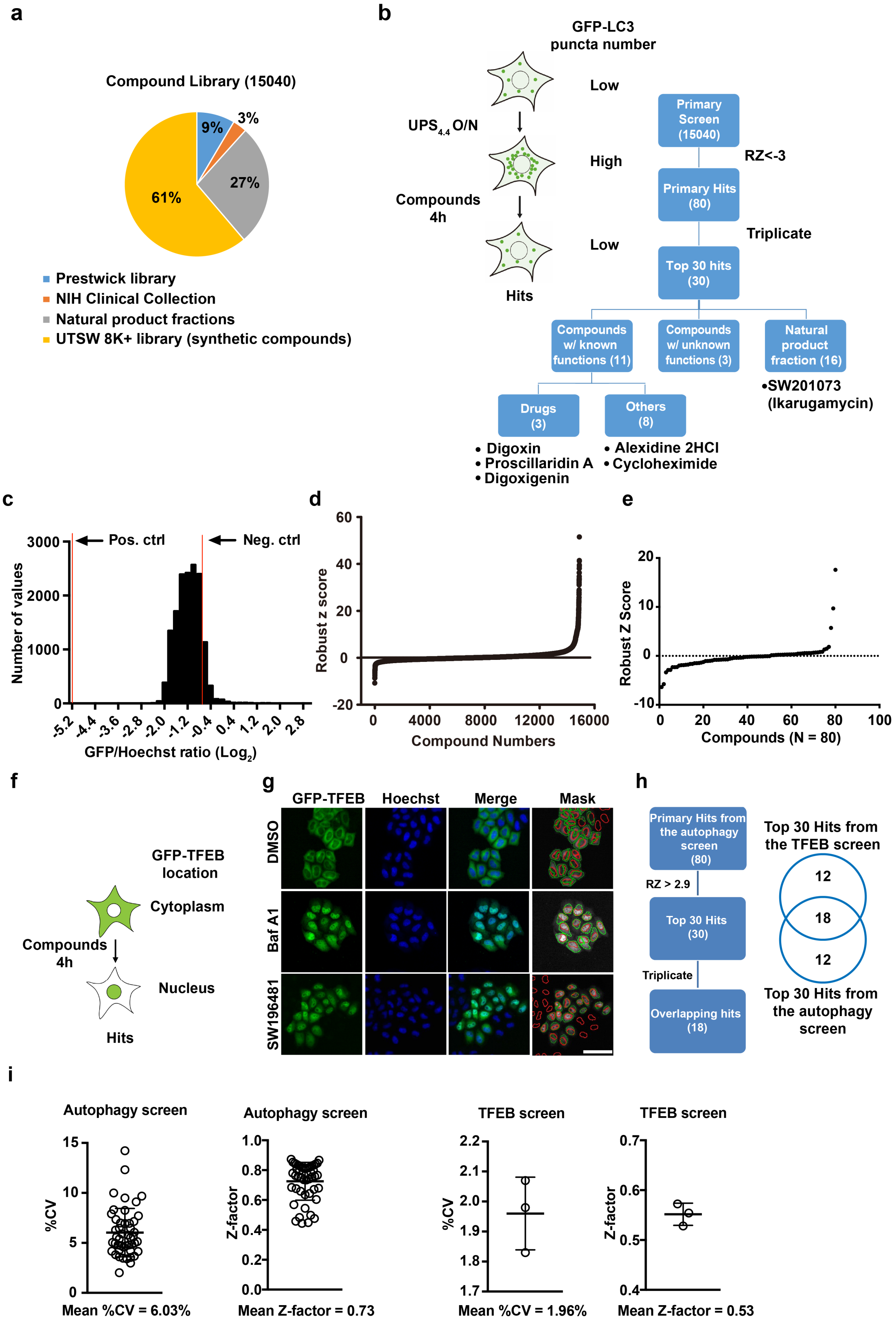

j

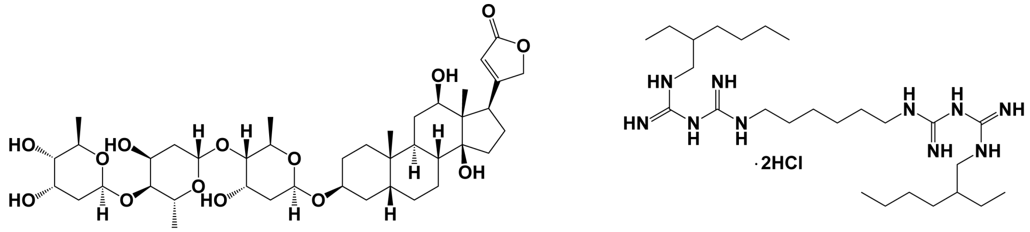

DG AD

k

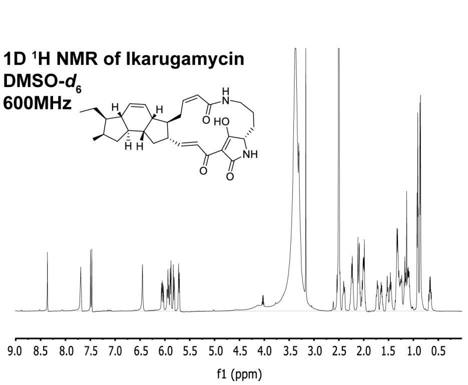

1D ¹H NMR of Ikarugamycin
DMSO-d6
600MHz
f1 (ppm)

l

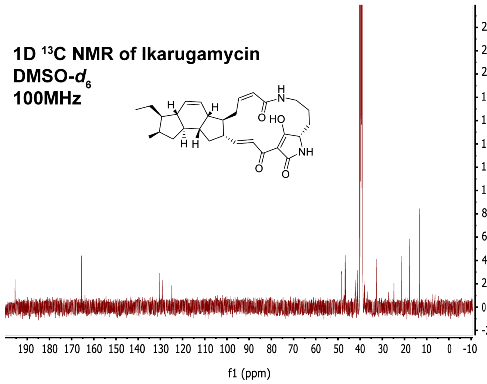

1D ¹³C NMR of Ikarugamycin
DMSO-d6
100MHz
f1 (ppm)

m

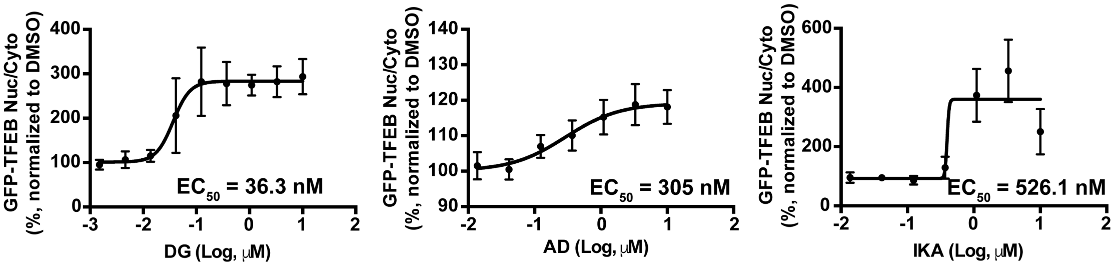

GFP-TFEB Nuc/Cyto (%, normalized to DMSO)
EC50 = 36.3 nM
DG (Log, μM)
EC50 = 305 nM
AD (Log, μM)
EC50 = 526.1 nM
IKA (Log, μM)

n

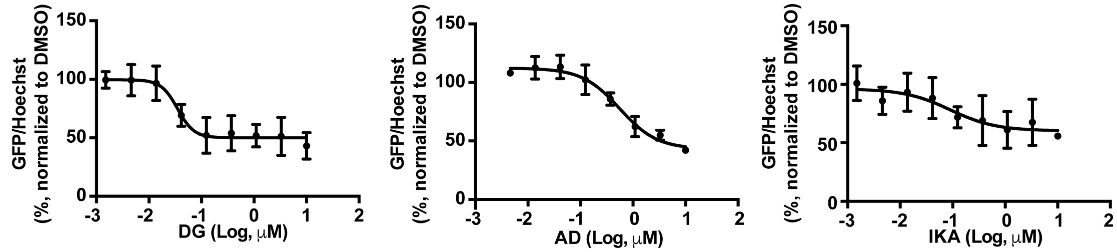

GFP/Hoechst (%, normalized to DMSO)
DG (Log, μM)
AD (Log, μM)
IKA (Log, μM)

o

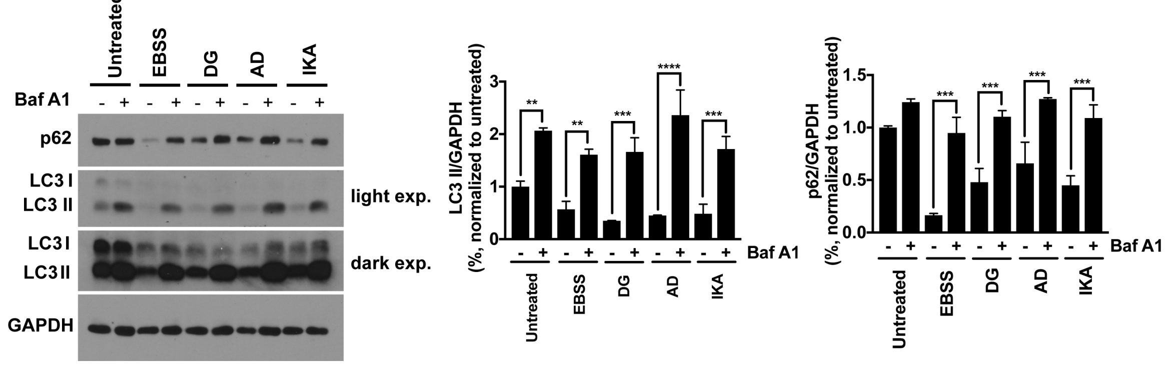

Untreated
EBSS
DG
AD
IKA
Baf A1
p62
LC3 I
LC3 II
light exp.
dark exp.
GAPDH
LC3 II/GAPDH (%, normalized to untreated)
p62/GAPDH (%, normalized to untreated)

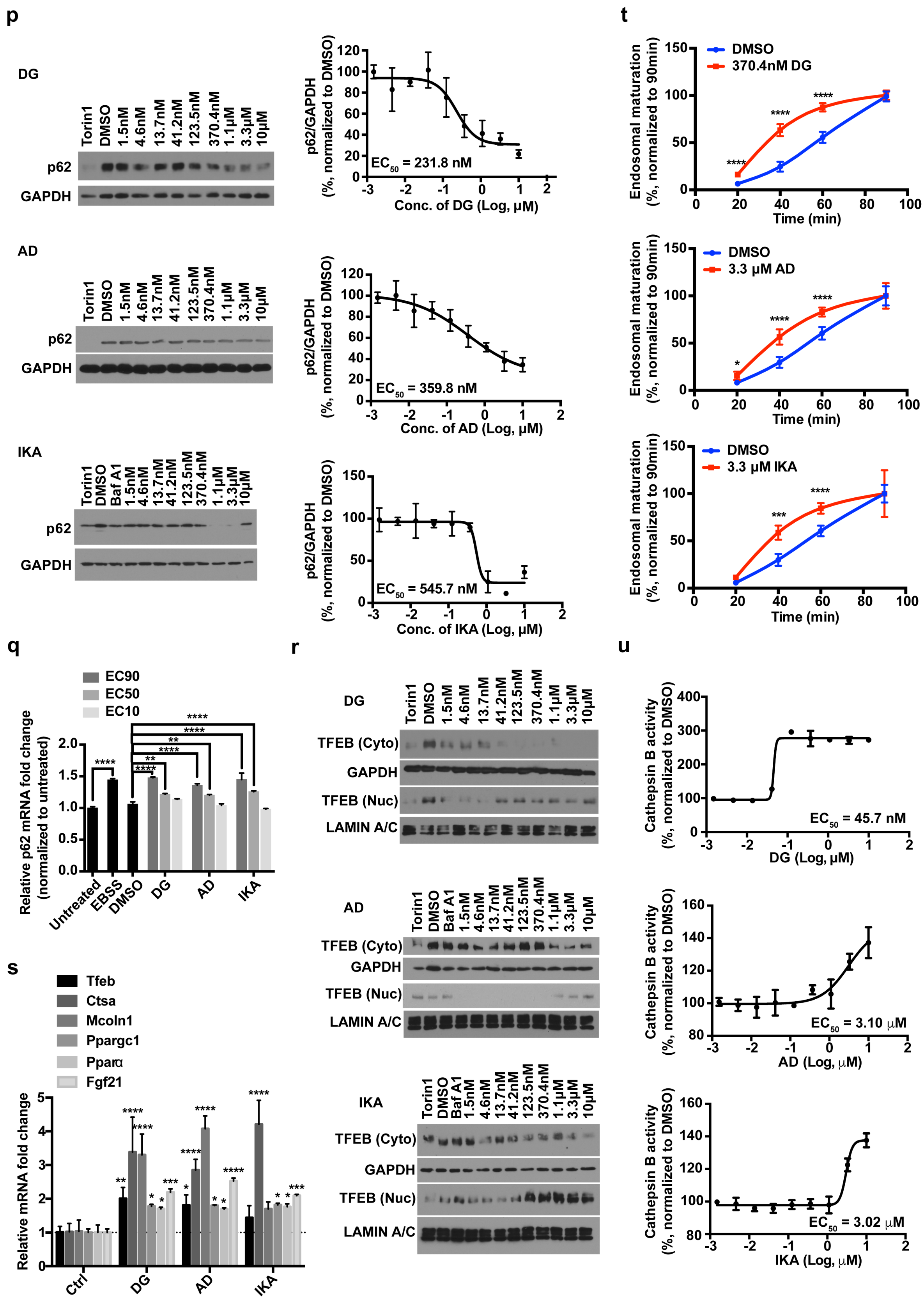

p
DG
Torin1 DMSO 1.5nM 4.6nM 13.7nM 41.2nM 123.5nM 370.4nM 1.1μM 3.3μM 10μM
p62
GAPDH
p62/GAPDH (%, normalized to DMSO)
EC50 = 231.8 nM
Conc. of DG (Log, μM)
AD
Torin1 DMSO 1.5nM 4.6nM 13.7nM 41.2nM 123.5nM 370.4nM 1.1μM 3.3μM 10μM
p62
GAPDH
p62/GAPDH (%, normalized to DMSO)
EC50 = 359.8 nM
Conc. of AD (Log, μM)
IKA
Torin1 DMSO Baf A1 1.5nM 4.6nM 13.7nM 41.2nM 123.5nM 370.4nM 1.1μM 3.3μM 10μM
p62
GAPDH
p62/GAPDH (%, normalized to DMSO)
EC50 = 545.7 nM
Conc. of IKA (Log, μM)
t
DMSO
370.4nM DG
Endosomal maturation (%, normalized to 90min)
Time (min)
DMSO
3.3 μM AD
Endosomal maturation (%, normalized to 90min)
Time (min)
DMSO
3.3 μM IKA
Endosomal maturation (%, normalized to 90min)
Time (min)
q
EC90
EC50
EC10
Relative p62 mRNA fold change (normalized to untreated)
Untreated EBSS DMSO DG AD IKA
r
DG
Torin1 DMSO 1.5nM 4.6nM 13.7nM 41.2nM 123.5nM 370.4nM 1.1μM 3.3μM 10μM
TFEB (Cyto)
GAPDH
TFEB (Nuc)
LAMIN A/C
AD
Torin1 DMSO Baf A1 1.5nM 4.6nM 13.7nM 41.2nM 123.5nM 370.4nM 1.1μM 3.3μM 10μM
TFEB (Cyto)
GAPDH
TFEB (Nuc)
LAMIN A/C
IKA
Torin1 DMSO Baf A1 1.5nM 4.6nM 13.7nM 41.2nM 123.5nM 370.4nM 1.1μM 3.3μM 10μM
TFEB (Cyto)
GAPDH
TFEB (Nuc)
LAMIN A/C
u
Cathepsin B activity (%, normalized to DMSO)
EC50 = 45.7 nM
DG (Log, μM)
Cathepsin B activity (%, normalized to DMSO)
EC50 = 3.10 μM
AD (Log, μM)
Cathepsin B activity (%, normalized to DMSO)
EC50 = 3.02 μM
IKA (Log, μM)
s
Tfeb
Ctsa
Mcoln1
Ppargc1
Pparα
Fgf21
Relative mRNA fold change
Ctrl DG AD IKA

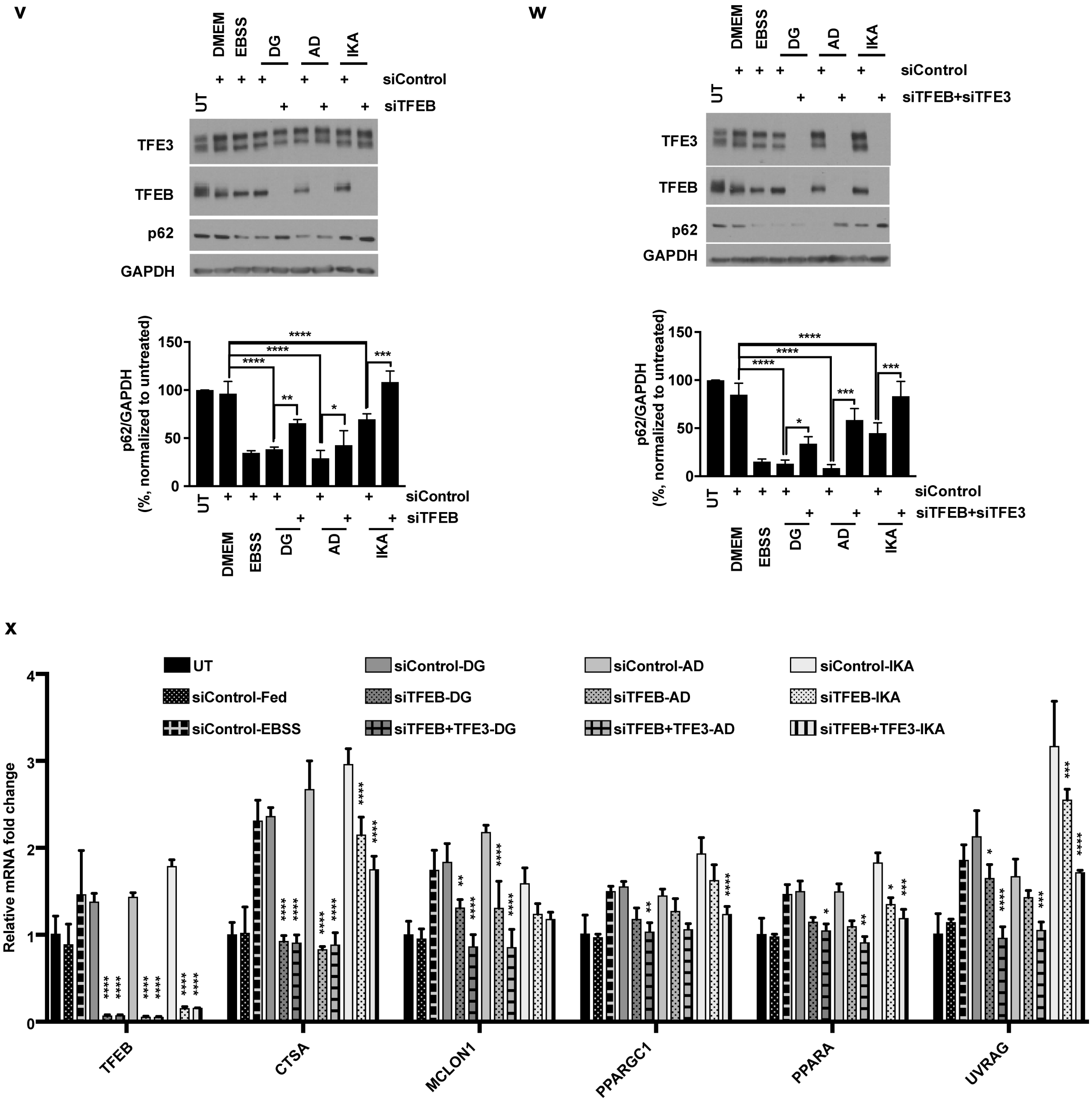

Supplementary Figure 2. A cell-based screen for small-molecule TFEB agonists. **(a)** A pie chart showing the composition of the chemical library. **(b)** Schematic of the autophagy screen and the workflow of it. **(c)** Distribution of all the screened chemicals over the GFP/Hoechst ratios after background correction. Two red lines indicate the positions of the positive control (wild-type HeLa cells) and the negative control (GFP-LC3 HeLa cells treated with $UPS_{4.4}$ only), respectively. **(d)** Robust Z score plot of all compounds in the autophagy screen. **(e)** Robust Z

score plot of the 80 primary hits of the autophagy screen in a triplicate confirmation assay. **(f)** Schematic of the TFEB screen. **(g)** Representative images from the high-content TFEB screen. The cells and their nucleus were identified and outlined in green and red, respectively. Bafilomycin A1 was used as a positive control in the screen. Scale bar, 50 μm. **(h)** Workflow of the TFEB screen and a schematic showing the overlapping compounds between the top 30 hit lists from the two screens. **(i)** The % CV (coefficient of variation) value and the Z-factor was calculated for all the plates of the autophagy (buffered with $UPS_{4.4}$, left) and TFEB screen (right), and the individual and averaged results were as shown. **(j)** Chemical structure of DG, and AD. **(k)** The ^{1}H NMR spectrum of purified IKA at 500 MHz in DMSO-d_6. **(l)** The ^{13}C NMR spectrum of purified IKA at 100 MHz in DMSO-d_6. **(m)** Dose-response curves of DG, AD and IKA in GFP-TFEB HeLa cells. **(n)** Dose-response curves of DG, AD and IKA in GFP-LC3 HeLa cells treated with $UPS_{4.4}$ showing the clearance of LC3 puncta by these compounds. **(o)** Autophagic flux and p62/SQSTM1 protein level changes were measured in HeLa cells treated with 370.4 nM DG, 3.3 μM AD and IKA in the presence or absence of 100nM baf A1 for 4 hr (mean ± s.d. for n = 2 independent experiments). Untreated cells in DMEM and nutrient-deprived cells in EBSS were used as controls. LC3-II/GAPDH (middle) and p62/GAPDH (right) ratios were quantified from immunoblots using ImageJ. **(p)** Western blot of wild-type HeLa cells treated with various doses of DG, AD and IKA (left panel). p62/SQSTM1 and GAPDH (glyceraldehyde-3-phosphate dehydrogenase) protein levels and the dose-response curves were quantified and simulated using ImageJ (mean ± s.d. for n = 2 independent experiments, right panel). **(q)** Quantitative polymerase chain reaction (qPCR) was used to quantify relative abundance of mRNA levels in HeLa cells treated with different doses (EC_{10}, EC_{50}, EC_{90}) of compounds. DMSO was used as a control (mean ± s.d. for n = 3 independent experiments, * $p<0.05$, *** $p<0.001$, **** $p<0.0001$). **(r)** Western blot of wild-type HeLa cells treated with various doses of DG, AD and IKA. Cytosolic and nuclear TFEB and the corresponding loading controls were blotted. **(s)** qPCR analysis result of MEFs treated with 370.4 nM DG, 3.3 μM AD and IKA for 4 hr (mean ± s.d. for n = 3 independent experiments, *

$p<0.05$, *** $p<0.001$, **** $p<0.0001$). **(t)** Endosomal maturation rate was measured in HeLa cells treated with DG, AD and IKA for 4 hr using 100 μg mL^{-1} always-ON/OFF-ON $UPS_{5.3}$ nanoprobes. **(u)** Dose-response curves of cathepsin B activity in cells treated with various doses of DG, AD and IKA. Immunoblots of wild-type HeLa cells under DG, AD and IKA treatment (EC_{90}) with control siRNA (siLONRF1), siTFEB or siTFEB in combination with siTFE3 are shown in the upper panel of (**v**) and (**w**). Corresponding quantification of p62 protein is shown in the lower panels of (**v**) and (**w**) (mean ± s.d., n = 2, * $p<0.05$, ** $p<0.01$, *** $p<0.001$, **** $p<0.0001$). **(x)** A qPCR analysis was done on HeLa cells under the same treatment conditions as used in (**v**) or (**w**). Significance testing between the siControl and siTFEB/siTFEB+siTFE3 groups in compound-treated cells was performed by two-way ANOVA (Tukey test, mean ± s.d., n = 3, * $p<0.05$, ** $p<0.01$, *** $p<0.001$, **** $p<0.0001$).

Supplementary Figure 3

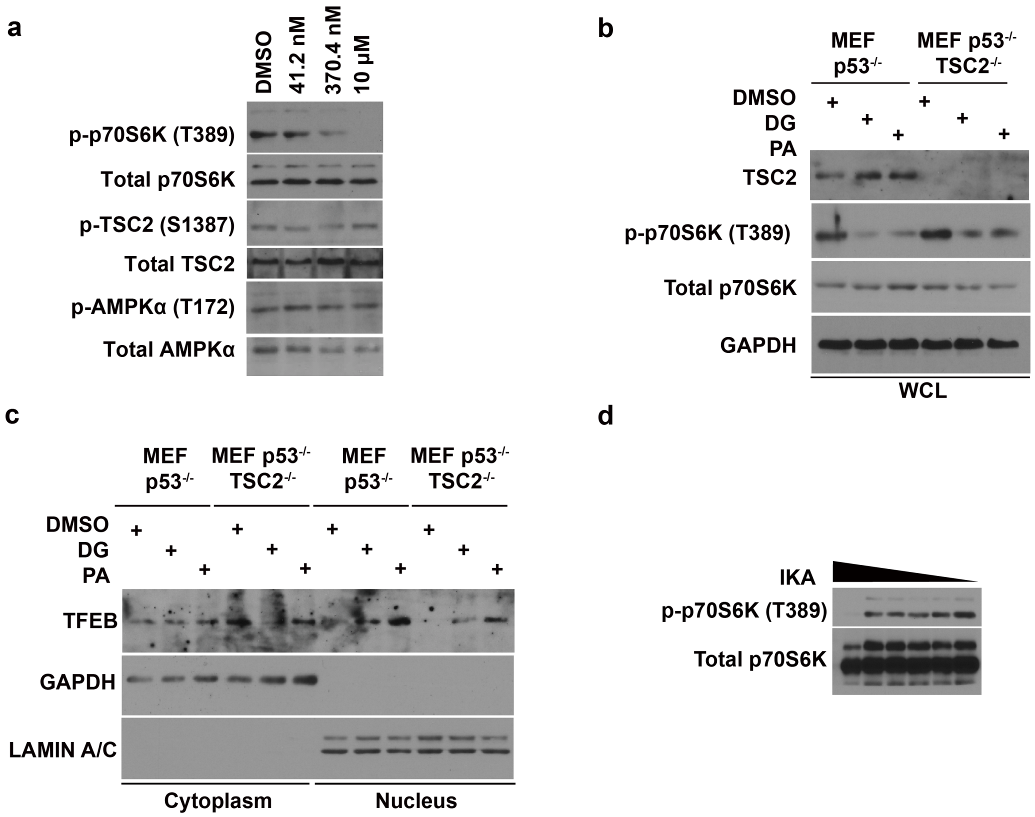

Supplementary Figure 3. DG and IKA inhibit the activity of mTORC1. **(a)** Immunoblots of the indicated proteins in HeLa cells treated with indicated doses of DG. **(b, c)** Immunoblots of *p53* $^{-/-}$ or *p53* $^{-/-}$ and *TSC2* $^{-/-}$ MEFs treated with 370.4 nM DG, proscillaridin A (PA) and DMSO using whole-cell-lysate (WCL) (**b**) and cytosolic/nuclear lysate (**c**). **(d)** Immunoblots of wide-type HeLa cells treated with various doses (starting from 3.3 μM with a 3-fold dilution, and rightmost lane is DMSO) of IKA. Phosphorylation of p70-S6 kinase (S6K) was used as the readout of mTORC1 activity.

Supplementary Figure 4

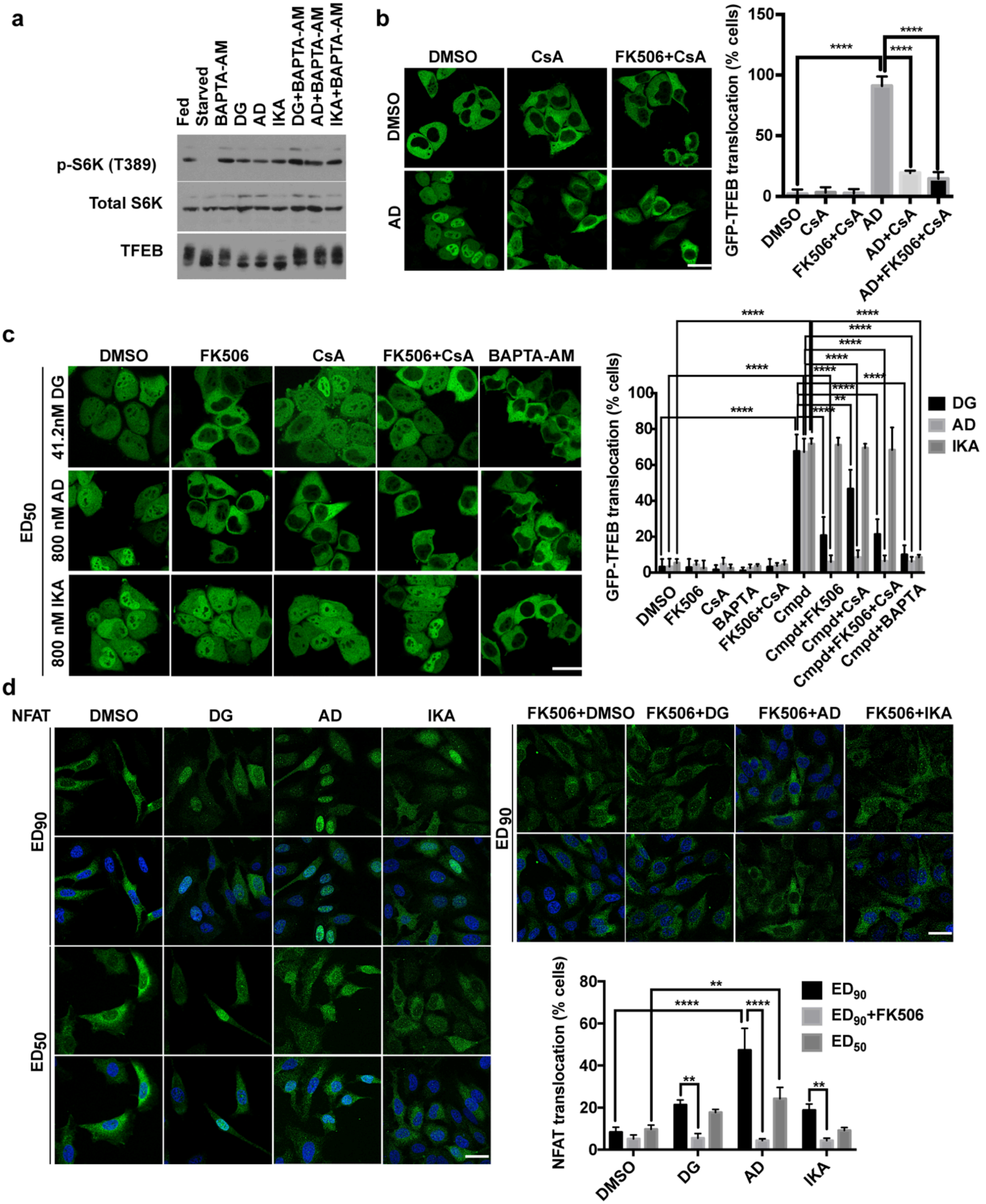

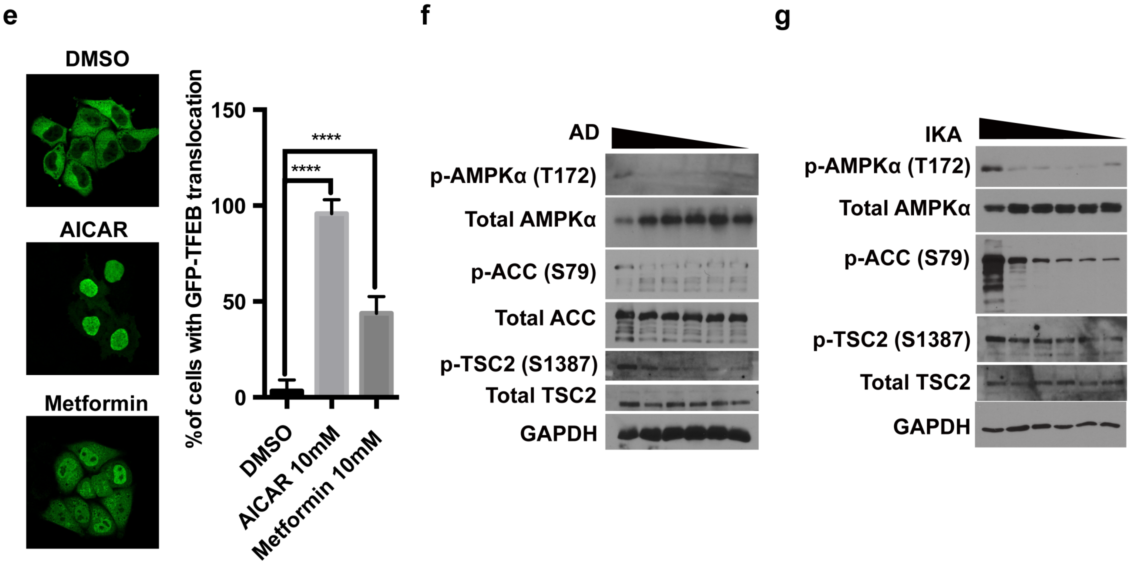

Supplementary Figure 4. Small-molecule agonists activate TFEB through different pathways. **(a)** HeLa cells were pretreated with or without 5 μM BAPTA-AM for 1 hr, washed off and treated with DG, AD and IKA for 2 hrs before lysates were collected. **(b)** Representative images of GFP-TFEB HeLa cells treated with 3.3 μM AD or in combination with 5 μM FK506, 10 μM CsA or both. The graph (right panel) represents the percentage of cells with GFP-TFEB translocation under these conditions (mean ± s.d. for n = 3 independent experiments, **** $p<0.0001$). Scale bar, 20 μm. **(c)** Representative images of GFP-TFEB HeLa cells treated with DG, AD and IKA at indicated intermediate doses (ED_{50}), and in combination with 5 μM BAPTA-AM, 5 μM FK506, 10 μM cyclosporine A (CsA) or both calcineurin inhibitors. The graph (right panel) represents the percentage of cells with GFP-TFEB translocation under these conditions (mean ± s.d. for n = 3 independent experiments, ** $p<0.01$, **** $p<0.0001$). Scale bar, 20 μm. **(d)** Endogenous NFAT nuclear translocation in cells treated with high (ED_{90}) or intermediate (ED_{50}) doses of DG, AD and IKA alone or with FK506. The graph shows the percentage of NFAT translocation (mean ± s.d. for n = 3 independent experiments, ** $p<0.01$, **** $p<0.0001$). **(e)** Known AMPK activators AICAR and metformin were sufficient to activate TFEB. The graph represents the percentage of cells with GFP-TFEB translocation under these conditions (mean ± s.d. for n = 3 independent experiments, **** $p<0.0001$). Scale bar, 20 μm. **(f, g)** Immunoblots of wide-type

HeLa cells treated with various doses (starting from 3.3 μM with a 3-fold dilution, and rightmost lane is DMSO) of AD and IKA.

Supplementary Figure 5

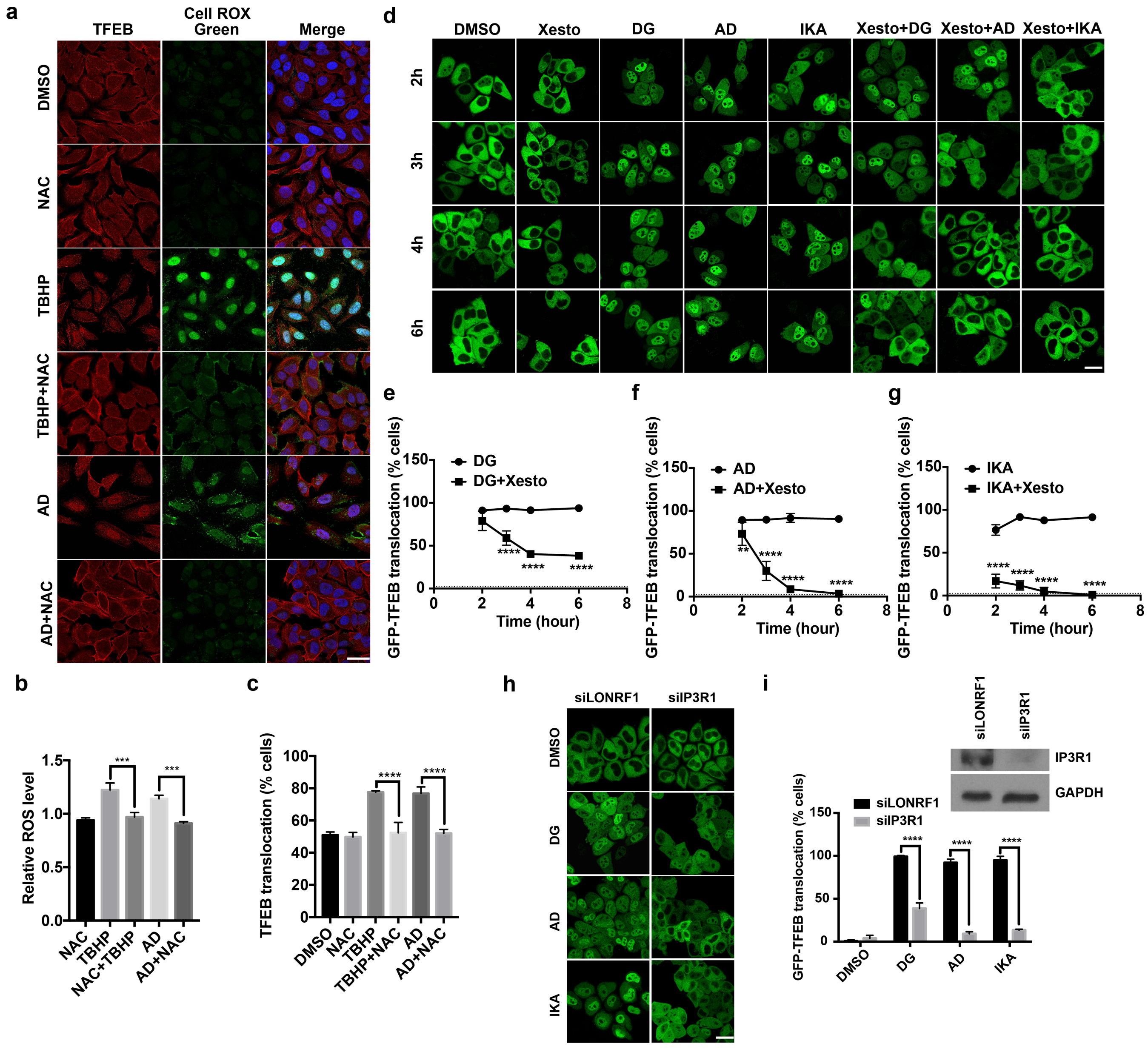

Supplementary Figure 5. Small-molecule activators of TFEB engage different sources of Ca^{2+}. **(a)** HeLa cells were pretreated with or without N-acetyl-cysteine (NAC) for 1 hr and then treated with tert-butyl hydroperoxide (TBHP) or AD for 4 hrs. TBHP and NAC were used as positive and negative controls. A ROS-sensitive DNA dye CellROX Green was used to detect cellular ROS levels after these treatments. Endogenous TFEB localization was also shown. **(b)** Quantification of the fluorescent intensity of CellROX Green in cells treated as in **a** was done by

a plate reader. **(c)** Quantification of endogenous TFEB nuclear translocation in **a**. **(d)** Cells were pretreated with or without 25 μM IP3R inhibitor Xestosporine C (Xesto) for 1 hr and then treated with DG, AD and IKA for indicated time. Quantification of TFEB translocation was shown in **e-g** (graphs represent mean ± s.d. ** $p<0.01$, *** $p<0.001$, **** $p<0.0001$). **(h, i)** RNA-interference were used to knock down inositol 145-trisphosphate receptor type 1 (IP3R1) in GFP-TFEB cells before they were treated with DG, AD and IKA. siLONRF1 was used as a negative control. TFEB translocation percentage was quantified from images as shown in (**i**) (mean ± s.d. for n = 3 independent experiments, **** $p<0.0001$). Scale bar, 20 μm.

Supplementary Figure 6

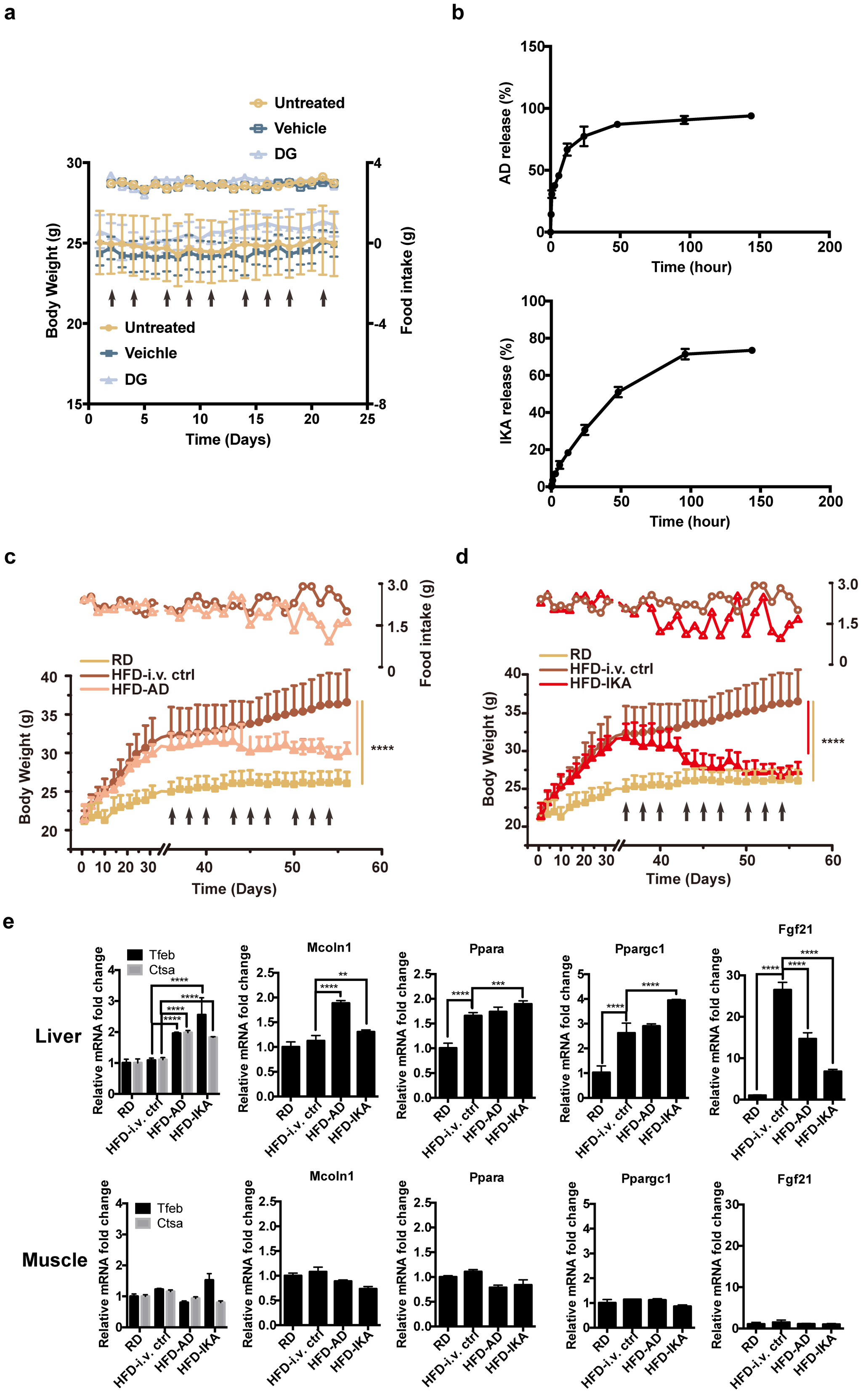

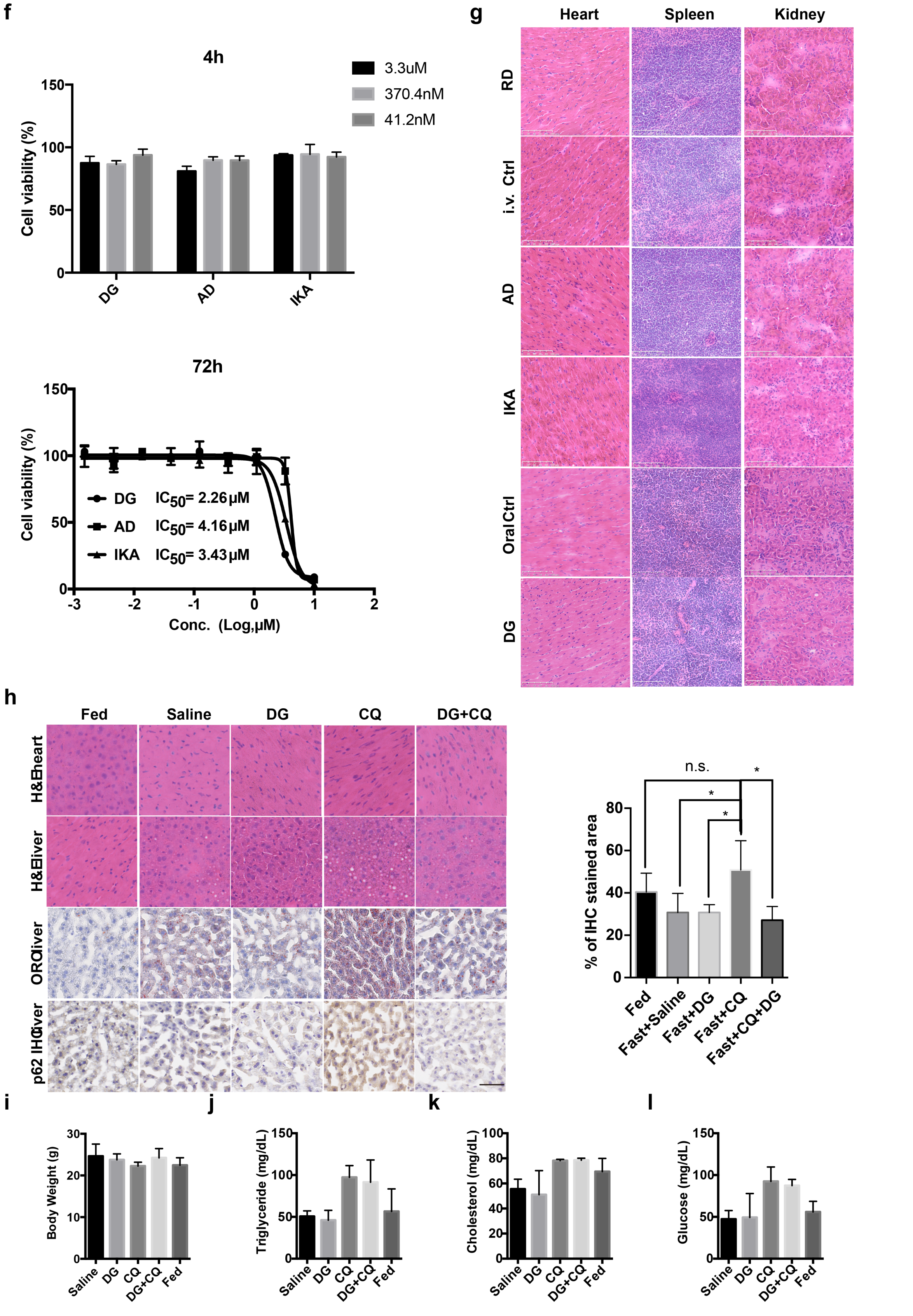
f
4h
3.3uM
370.4nM
41.2nM
Cell viability (%)
DG
AD
IKA
72h
DG IC50= 2.26μM
AD IC50= 4.16μM
IKA IC50= 3.43μM
Conc. (Log,μM)
g
Heart
Spleen
Kidney
RD
i.v. Ctrl
AD
IKA
Oral Ctrl
DG
h
Fed
Saline
DG
CQ
DG+CQ
H&E Heart
H&E Liver
ORO Liver
p62 IHC Liver
n.s.
% of IHC stained area
Fed
Fast+Saline
Fast+DG
Fast+CQ
Fast+CQ+DG
i
j
k
l
Body Weight (g)
Triglyceride (mg/dL)
Cholesterol (mg/dL)
Glucose (mg/dL)
Saline
DG
CQ
DG+CQ
Fed

Supplementary Figure 6. Small–molecule activators of TFEB decrease the body weight of fat mice without induce toxicity in major organs, and improved acute lipid-accumulation induced by short-term starvation and chloroquine (CQ) treatment. **(a)** The body weight (left y-axis, solid symbols) and food intake (right y-axis, open symbols) was measured in normal-diet-fed mice treated with vehicle or 2.5mg/kg DG. **(b)** Drug release curves of AD (upper panel) and IKA (lower panel) in PBS. **(c, d)** Food uptake (open symbols) and body weight changes (solid symbols) of mice feed with regular diet (RD), high-fat diet with intravenous injection of empty PEG-PLA nanoparticles (HFD-i.v. ctrl) and HFD with AD (HFD-AD) or IKA (HFD-IKA) i.v. injections three times a week starting from Day 35 as indicated by the arrows (bars represent mean ± s.d. * $p<0.05$, ** $p<0.01$, *** $p<0.001$, ****$p<0.0001$, HFD-AD or HFD-IKA compared with HFD-i.v. ctrl group). **(e)** Known TFEB target genes were upregulated in the liver rather than in the muscle of HFD mice treated with AD or IKA. qPCR analysis of mRNA levels of some known TFEB target genes, including known TFEB targets *Tfeb*, *Csta* and *Mcoln1*, and key regulators of lipid metabolism *Ppargc1α*, *Ppar1α*and *FGF21* in the liver (upper panel) and muscle (lower panel) samples from mice treated with AD, IKA or their corresponding controls (HFD-i.v. ctrl). **(f)** Cells were treated with various doses DG, AD and IKA for 4 hours, and cell viability was measured immediately or after 72 hours. Dose response curves were simulated using ImageJ. **(g)** H&E staining of heart, spleen and kidney sections from mice treated with DG, AD, IKA and their corresponding controls. Scale bar, 100 μm. **(h)** H&E staining of liver and heart sections as well as ORO and p62/SQSTM1 IHC staining of liver sections from mice under fed and fast conditions with orally injected DG and intraperitoneally injected CQ, individually and jointly, or their corresponding control (Saline). Scale bar, 50 μm. **(i)** The body weight of mice after the treatment described in **h**. **(i-k)** Total serum levels of cholesterol, triglyceride and glucose of mice after the treatment described in **g**.

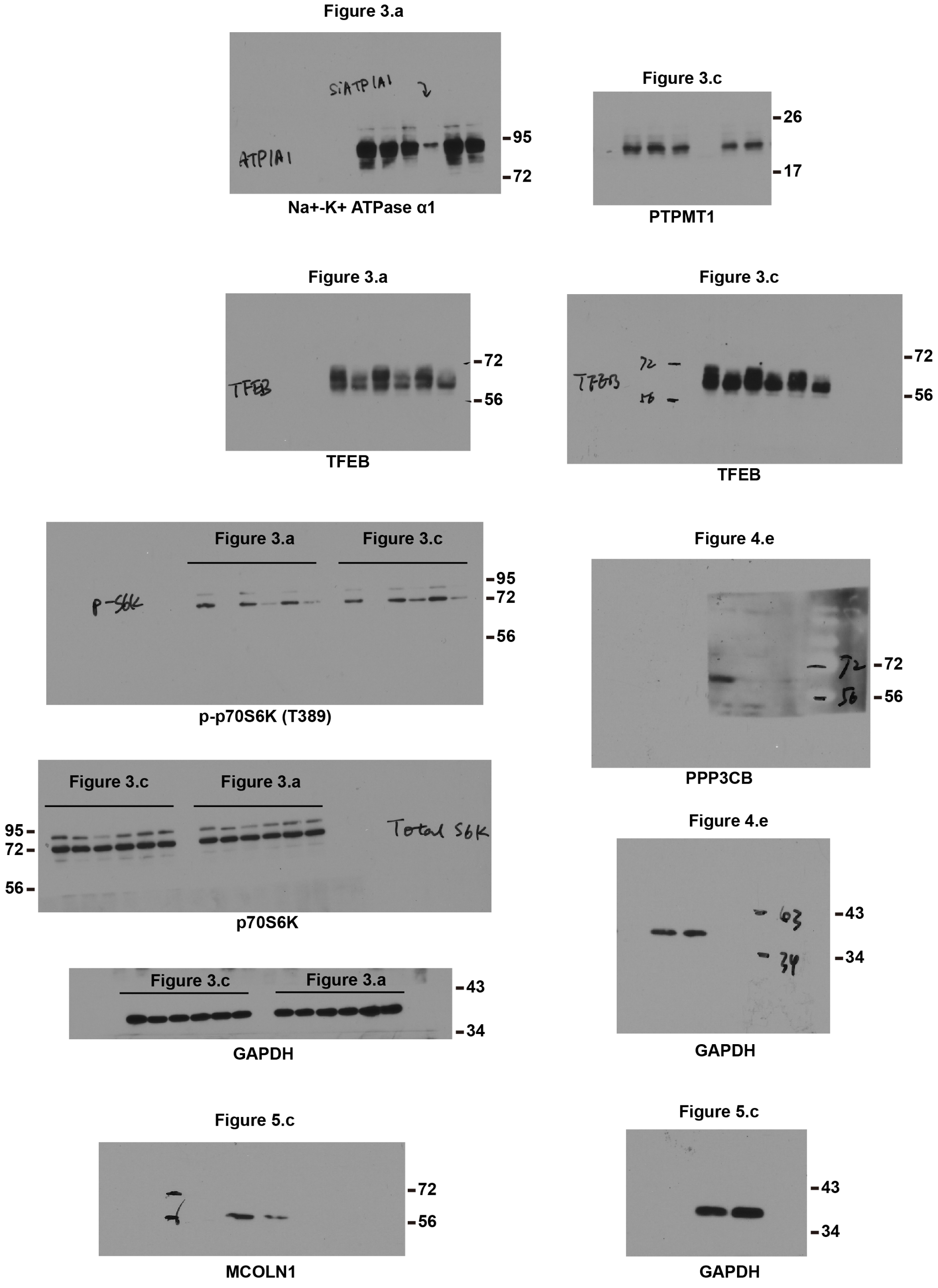

Supplementary Figure 7. The original blots that correspond to those presented in the main paper.

Supplementary Table 1. Characterization of AD- and IKA-loaded PEG_{5000}-PLA_{5000} nanoparticles.

	PEG-PLA	PEG-PLA+AD	PEG-PLA+IKA
**D_h (nm)[a]**	67.9	68.1	68.2
**PDI[b]**	0.168	0.172	0.15
**Loading content (%wt)**	N/A	5.4	3.5

[a, b]The hydrodynamic diameter (D_h) and polydispersity index (PDI) were analyzed by dynamic light scattering analysis.

Supplementary Table 2. Statistical analysis of the lifespan experiments.

Compound treatment		Mean Lifespan ± s.e.m. (Median lifespan in days)	75th% (Day)	Observed /Total events	Lifespan increase (%)	p-values
**1st**	**DMSO**	18.5±0.5 (18)	22	98/100	N/A	N/A
	**IKA**	21.8±0.6 (22)	26	97/100	22.2	<0.001
**2nd**	**DMSO**	18.8±0.5 (17)	22.5	80/80	N/A	N/A
	**IKA**	23.0±0.5 (24)	26	79/80	22.3	<0.001
